# K2P channels as emerging therapeutic targets for pain: insights from functional studies and transcriptomic analyses

**DOI:** 10.3389/fphar.2026.1907761

**Published:** 2026-09-03

**Authors:** Nicolas Gilbert, Kelly Jeanne, Athena Li, Thomas Lorivel, Franck C. Chatelain, Florian Lesage, Delphine Bichet

**Affiliations:** Institut de Pharmacologie Moléculaire et Cellulaire (IPMC), Inserm, CNRS, Université Côte d’Azur, Valbonne, France

**Keywords:** analgesic targets, ion channels, neuronal excitability, nociception and pain, pain models, sensory neurons, two-pore domain potassium channels (K2P channels)

## Abstract

Two-pore domain potassium (K2P) channels are major determinants of neuronal excitability through their ability to generate leak potassium currents that control resting membrane potential and action potential firing. Initially considered as passive background channels, K2P channels are now recognized as polymodal sensors integrating mechanical, thermal, chemical, inflammatory, and metabolic signals. Over the past two decades, accumulating evidence has revealed their essential contribution to nociceptive processing and their potential as therapeutic targets for pain disorders. In this review, we provide an integrated overview of K2P channel function in pain, combining data from anatomical, transcriptomic, electrophysiological, genetic, behavioral, and pharmacological studies. We first summarize recent advances defining K2P channel expression throughout the nociceptive system, from peripheral sensory neurons to spinal and supraspinal circuits. Integration of large-scale single-cell transcriptomic datasets from rodents and humans reveals that most K2P members, beyond the historically studied TREK1 and TRESK channels, are expressed in pain-related pathways with distinct cell-type and species-specific patterns. We then discuss how K2P channel expression and function are altered in pathological conditions, including inflammatory, neuropathic, cancer-associated, chemotherapy-induced, migraine-related pain states. Genetic loss-of-function studies consistently demonstrate that reduced K2P activity increases neuronal excitability and promotes pain hypersensitivity, whereas pharmacological activation of selected K2P channels restore inhibitory control and produce analgesic effects. Rather than supporting the existence of a single universal K2P “pain channel”, current evidence suggests that different K2P subtypes contribute to specific sensory modalities, neuronal populations, and pathological contexts. Future therapeutic strategies will therefore require identification of the most relevant K2P targets according to pain mechanisms and benefit/risk profiles. Together with advances in structural biology and drug discovery, these findings position K2P channels as a versatile family of targets for precision analgesia and future multimodal treatments aimed at restoring the balance between excitatory and inhibitory mechanisms in chronic pain.

## 1 Introduction

### 1.1 From leak currents to polymodal K2P channels

Two-pore domain potassium (K2P) channels constitute a distinct family of potassium (K^+^) channels, commonly referred to as “leak” or “background” K^+^ channels because of their major contribution to resting membrane conductance. The concept of a K^+^ current largely independent of voltage and time was first introduced by Hodgkin and Huxley in 1952 through recordings from the squid giant axon (Hodgkin and Huxley, 1952). However, the molecular identity of channels underlying such currents remained elusive for several decades.

The first protein associated with a background potassium conductance was identified in yeast with the cloning of the **TOK** channel (Tandem-pore Outward rectifying K^+^ channel), although this channel displays notable structural and functional differences from mammalian K2P channels (Ketchum et al., 1995). The first mammalian K2P channel was identified shortly thereafter with the cloning of *KCNK1*, reported by the group of Michel Lazdunski and named **TWIK1** (for Tandem of Weak Inwardly rectifying K^+^ channel) (Lesage et al., 1996a). This discovery initiated the isolation of many related channels. Between 1996 and 2003, a total of 15 K2P genes were identified in human (*KCNK1–KCNK*18) and rodents as well as numerous isoforms (Goldstein et al., 2001; Lesage and Lazdunski, 2000; Niemeyer et al., 2016; Enyedi and Czirják, 2010).

On the basis of sequence homology and distinctive regulatory features, these channels are currently grouped into six subfamilies (common name in bold, human gene in italic and channel name in another nomenclature):

- **TREK** (TWIK-related K^+^ channel) and **TRAAK** (TWIK-Related Arachidonic Acid-stimulated K^+^ channel) channels, including **TREK1** (*KCNK2*, K2P2.1), **TREK2** (*KCNK10*, K2P10.1), and **TRAAK** (*KCNK4*, K2P4.1);
- **TALK** (TWIK-related ALkaline pH-activated K^+^ channel) channels, comprising **TALK1** (*KCNK16*, K2P16.1), **TALK2** (*KCNK17*, K2P17.1), and **TASK2** (*KCNK5*, K2P5.1);
- **TWIK** channels, including **TWIK1** (*KCNK1*, K2P1.1), **TWIK2** (*KCNK6*, K2P6.1), and **TWIK3** (*KCNK7*, K2P7.1);
- **THIK** (Tandem-pore domain Halothane-Inhibited K^+^ channel) channels, composed of **THIK1** (*KCNK13*, K2P13.1) and **THIK2** (*KCNK12*, K2P12.1);
- **TASK** (TWIK-related Acid-Sensitive K^+^ channel) channels, including **TASK1** (*KCNK3*, K2P3.1), **TASK3** (*KCNK9*, K2P9.1), and **TASK5** (*KCNK15*, K2P15.1);
- **TRESK** (TWIK-RElated Spinal cord K^+^ channel) channel, represented by only one member **TRESK** (*KCNK18*, K2P18.1).

K2P channels are defined structurally by the presence of two pore-forming loops (P-loops) per subunit, distinguishing them from the other major K^+^ channel families, KV (voltage-gated potassium channel) and KIR (inwardly rectifying potassium channel), which contain one pore loop per subunit. Functional K2P channels assemble as dimers, each subunit comprising four transmembrane segments and two pore loops that together form a single K^+^-selective conduction pathway. Like all K^+^ channels, their selectivity filter contains a highly conserved signature sequence responsible for K^+^ selectivity.

Although not gated by voltage, K2P channels do not behave as passive pores. Instead, their activity can be affected by membrane potential resulting to some extent in either outward or inward rectifications (Enyedi and Czirják, 2010). Beyond this intrinsic voltage sensitivity, K2P channels are subject to regulation by a wide range of physical and chemical stimuli, including mechanical stretch, temperature, membrane lipids, extracellular and intracellular pH, neurotransmitters, and multiple GPCR (G protein-coupled receptor)-dependent signaling pathways involving Gαq, Gβγ, PKA/PKC (protein kinase A/C), and PLC (phospholipase C) (Feliciangeli et al., 2015; Mathie et al., 2010).

By integrating diverse physicochemical and signaling inputs, the polymodal regulation of K2P channels positions them as central hubs for the integration of biological signals and the fine-tuning of cellular excitability.

### 1.2 Physiological roles of K2P channels beyond nociception

Beyond nociception, which is discussed in detail in the following sections of this review, K2P channels play essential roles in a broad spectrum of physiological processes.

K2P channels, particularly **TASK** channels, play a pivotal role in respiratory physiology by contributing to central respiratory rhythm generation and sleep–wake regulation through oxygen and carbon dioxide sensing in the carotid body and brainstem chemoreceptors. In addition, they modulate upper airway tone and hypoxia-induced pulmonary vasoconstriction (Lin et al., 2025). Consistent with these functions, mice lacking **TASK** channels exhibit blunted ventilatory responses to both central and peripheral hypoxia, as well as altered systemic and pulmonary blood pressure regulation (Kim and Kang, 2015). In humans, loss-of-function (LOF) mutations in **TASK1** have been linked to familial and idiopathic pulmonary arterial hypertension, whereas gain-of-function (GOF) mutations cause sleep-disordered breathing, including central sleep apnea (Duprat et al., 1997; Gestreau et al., 2010; Girerd et al., 2016; Gurney et al., 2003; Ma et al., 2013; Navas Tejedor et al., 2017; Olschewski et al., 2006; Simonson et al., 2022; Sörmann et al., 2022). **TWIK2** knockout (KO) mice also exhibit systemic and pulmonary hypertension, suggesting a role for this channel in the regulation of vascular contractility (Lloyd et al., 2011; Pandit et al., 2014). In the heart, several K2P channels—including **TREK1**, **TWIK1**, **TASK1**, and **TALK2**—contribute to cardiac action potential and atrial excitability, thereby influencing susceptibility to arrhythmias (Ma et al., 2013; Schmidt et al., 2015). In zebrafish, genetic ablation of **TWIK1** (which is absent in the mouse heart) results in bradycardia and atrial dilation, highlighting a conserved role in cardiac rhythm control (Christensen et al., 2016). **TWIK1** also displays dynamic ion selectivity under conditions of hypokalemia and acidosis, a distinctive property compatible with a role in paradoxical depolarization during hypokalemic states (Chatelain et al., 2013; Chen et al., 2014; Ma et al., 2011; Chatelain et al., 2024). Although the different cardiac K2P channels fulfill distinct functions in the heart, most are expressed in cardiomyocytes and pacemaker cells and are recognized as contributors to cardiovascular functions (Herrera-Pérez et al., 2021; Wiedmann et al., 2021). Consistently, pathogenic variants in human **TALK2** and **TREK1** have been associated with tachycardia, idiopathic ventricular fibrillation, and long QT syndrome (Chai et al., 2018; Decher et al., 2017; Friedrich et al., 2014).

In endocrine tissues, **TALK1** and **TALK2** regulate glucose-stimulated insulin secretion in pancreatic β-cells, and GOF mutations in **TALK1** have been associated with human MODY (maturity-onset diabetes of the young)-like diabetes (Graff et al., 2021; Tsai et al., 2022; Vierra et al., 2015; Vierra et al., 2017). Because these channels can heteromerize in β-cells, their functional effects are mediated by both homomeric and heteromeric channel assemblies (Duprat et al., 2005; Khoubza et al., 2022). In addition, **TASK1** and **TASK2** are also expressed in the pancreas and contribute to the physiology of both endocrine and exocrine pancreatic cells (Cid et al., 2013; Dadi et al., 2014). In adrenal gland, **TREK** and **TASK** are involved in stimulus-secretion of aldosterone in response to stress, hypoxia or metabolic cues (Kim and Kang, 2015; Bandulik et al., 2010; Davies et al., 2008; Enyeart et al., 2012; Guagliardo et al., 2019; Penton et al., 2012). **TASK1** also plays a role in the functional zonation of the adrenal gland (Heitzmann et al., 2008). K2P channels also play key roles in the regulation of immune cell function, with **THIK1** emerging as a major regulator in microglia, where it governs cellular surveillance, process ramification, phagocytic activity, and cytokine release (Kim et al., 2026; Madry et al., 2018). **TWIK2** has also been identified as a regulator of K^+^ flux in macrophages, a mechanism that is essential for the establishment of NLRP3 (NOD-(nucleotide-binding oligomerization domain), LRR-(leucine-rich repeat) and pyrin domain-containing protein 3)-dependent inflammasome activation in response to stimuli such as LPS (lipopolysaccharide) or ATP (adenosine triphosphate) (Di et al., 2018; Yan et al., 2022; Khanra et al., 2025). Accordingly, inhibition of **TWIK2** leads to reduced inflammasome activation (Yan et al., 2022). In addition, other K2P channels, including **TASK2**, **TASK1**/**TASK3** and **TREK1**, are expressed in lymphocytes and macrophages, where they influence immune responses. K2P channels also contribute to renal function and acid–base homeostasis, with **TASK2** playing a key role in bicarbonate reabsorption and cell volume regulation, and **TWIK1** participating in water and phosphate reabsorption (Barriere et al., 2003; Nie et al., 2005; Niemeyer et al., 2001; Warth et al., 2004). More broadly, several K2P channels have been implicated in the regulation of apoptosis, cell proliferation, migration, and mitochondrial function, underscoring their fundamental and ubiquitous roles in both cellular homeostasis and systemic physiology (Cikutović-Molina et al., 2019; Lauritzen et al., 2005; Lauritzen et al., 2003; Leithner et al., 2016).

In the nervous system, K2P channels contribute to the control of resting membrane potential (RMP) and input resistance, thereby shaping neuronal firing patterns, spike frequency and overall network excitability (Enyedi and Czirják, 2010). Owing to their sensitivity to a wide array of physical and chemical stimuli, they couple neuronal activity to metabolic status and environmental conditions, influencing synaptic integration and network oscillations, while also conferring neuroprotection in settings of ischemia, hypoxia, or excessive neuronal activity. Beyond these fundamental roles, several members of the K2P family are involved in higher-order brain functions, including the regulation of mood, stress responses, and anesthesia. In particular, **TREK1**—modulated by the antidepressant fluoxetine—has been studied in the context of depression and anxiety, whereas **TASK1** and **TASK3** channels, as major molecular targets of volatile anesthetics, play key roles in cognition, sleep, sedation, hypnosis, and general anesthesia (Heurteaux et al., 2006; Honoré, 2007; Lazarenko et al., 2010; Patel et al., 1999). **TRAAK** and **TASK3** channels have been identified as regulators of brain metabolism, particularly under ischemic conditions in mice (Ehling et al., 2010; Laigle et al., 2012). In humans, GOF mutations in **TRAAK** have been reported in several families affected by a complex neurodevelopmental disorder known as FHEIG syndrome, an acronym referring to its hallmark features: facial dysmorphism (F), hypertrichosis (H), epilepsy (E), intellectual disability (I), and gingival overgrowth (G) (Bauer et al., 2018). Several mutations in the **TASK3** channel have also been identified as the cause of a rare but severe neurodevelopmental disorder, the Birk–Barel syndrome (Barel et al., 2008). These findings point to a broader role for **TRAAK** and **TASK3** channel activity in central nervous system (CNS) development and function.

## 2 Expression of K2P channels in pain-related structures

### 2.1 Definitions and pain-related structures

**FIGURE 1:**
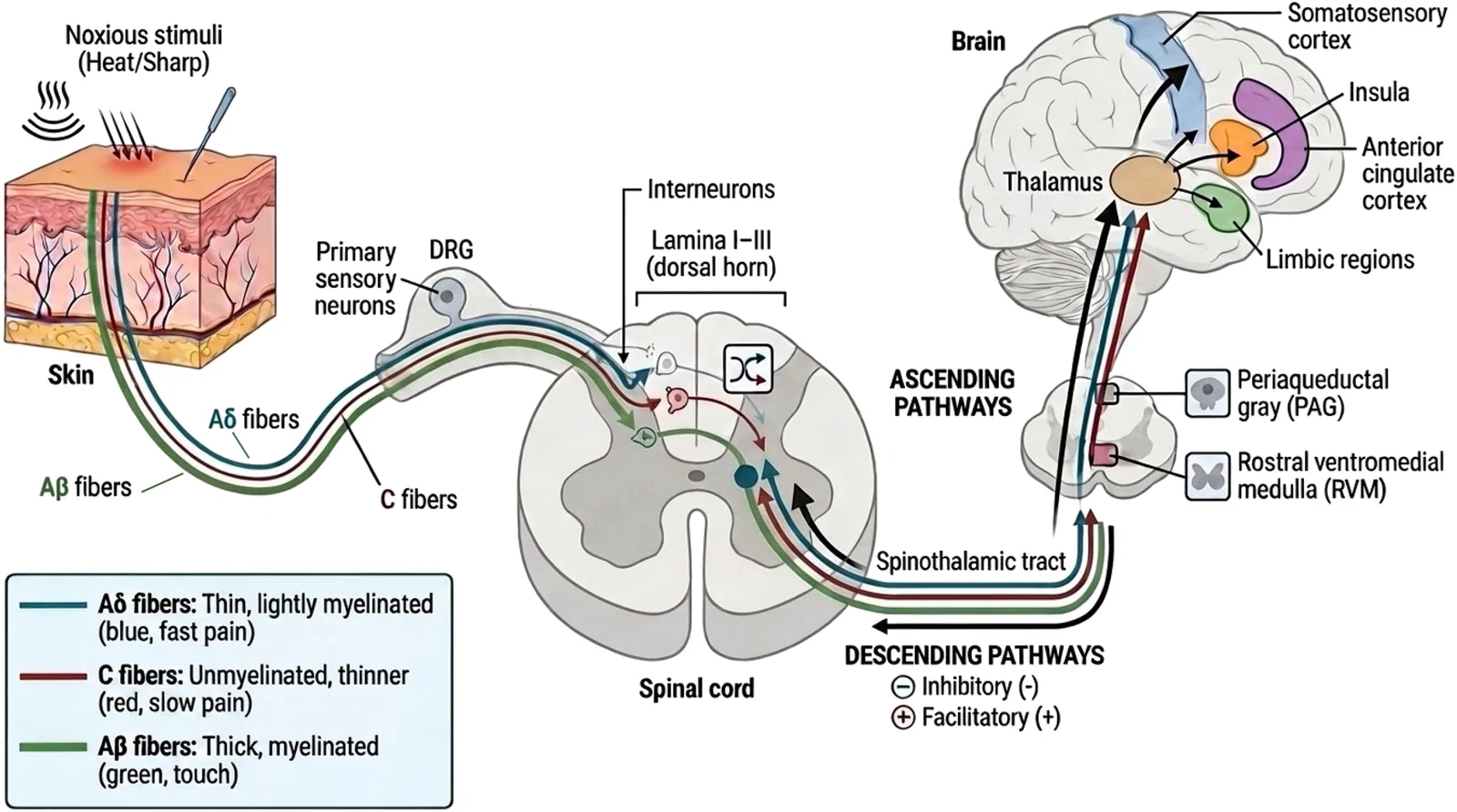
Schematic representation of pain perception pathways. Noxious stimuli are detected by primary sensory neurons located in dorsal root ganglia (DRG) or trigeminal ganglia (TG) and transmitted through distinct sensory fibers: Aδ fibers convey rapid, sharp pain, C fibers mediate slow and diffuse nociceptive signals, whereas Aβ fibers transmit innocuous touch. Primary afferents project to the dorsal horn of the spinal cord, where nociceptive information is integrated by local neuronal circuits before being transmitted through ascending pathways to brain regions involved in sensory and affective pain perception, including the thalamus, somatosensory cortex, insula, anterior cingulate cortex, and limbic structures. Descending pathways from supraspinal centers, including the periaqueductal gray (PAG) and rostral ventromedial medulla (RVM), provide inhibitory and facilitatory modulation of spinal nociceptive processing.

Pain perception involves complex network of peripheral and central structures that detect, transmit, and modulate nociceptive signals in both rodents and humans (Julius and Basbaum, 2001). At the peripheral level, noxious stimuli are detected by specialized primary sensory neurons whose cell bodies reside in the dorsal root ganglia (DRG) or trigeminal ganglia (TG). These neurons innervate peripheral tissues such as the skin, muscles, and viscera and project centrally to the spinal cord (Figure 1).

Nociceptive information is primarily conveyed by two major classes of sensory fibers: thinly myelinated Aδ fibers, which mediate fast and sharp pain, and unmyelinated C fibers, which transmit slower, diffuse and persistent pain. In contrast, large myelinated Aβ fibers mainly convey innocuous mechanical stimuli but can contribute to pain signaling under pathological conditions (Julius and Basbaum, 2001). In the spinal cord, primary afferents synapse onto second-order neurons and interneurons in the dorsal horn, particularly within laminae I–III, where nociceptive signals are integrated and modulated (Todd, 2010). These signals are then relayed through ascending pathways, such as the spinothalamic tract, to supraspinal structures including the thalamus, somatosensory cortex, insula, anterior cingulate cortex, and limbic regions, which together contribute to the sensory-discriminative and affective dimensions of pain (Todd, 2010; Price, 2000). In parallel, descending pathways originating from brainstem regions such as the periaqueductal gray and the rostral ventromedial medulla exert inhibitory or facilitatory control over spinal nociceptive transmission (Ossipov et al., 2010). Although the general organization of these circuits is highly conserved between rodents and humans, species-specific differences in fiber composition, receptor expression, and circuit organization exist (Rostock et al., 2018).

K2P channels are widely expressed throughout the nociceptive system, spanning peripheral nociceptors, DRG, TG, spinal dorsal horn laminae, ascending spinothalamic pathways, thalamic relay nuclei, and cortical regions including S1/S2, the insula, and the anterior cingulate cortex (Huang et al., 2022; Pollema-Mays et al., 2013; Rosner et al., 2023; Sarıtaş et al., 2025; Smith, 2020). Despite this broad distribution, establishing a detailed and unified expression atlas remains challenging. Since the early 2000s, *in situ* hybridization, qPCR (quantitative polymerase chain reaction), bulk transcriptomics, and immunohistochemistry have substantially advanced our understanding of K2P expression patterns in rodent central- and peripheral nervous systems (CNS and PNS) (Lesage et al., 2000; Lesage et al., 1996b; Marsh et al., 2012; Messina et al., 2022; Moayedi and Davis, 2013). While these approaches laid the groundwork for identifying cell- and region-specific expression, a major leap forward occurred with the 2024 large-scale meta-analysis by Bhuiyan et al. (2024), which integrated 15 independent mouse RNA-sequencing datasets (Drop-seq, inDrops, single molecule real-time (SMRT) sequencing, 10x Genomics 3′RNA) and 3 human datasets (10 × 3′RNA and Visium). This comprehensive integration provides the most robust overview to date of K2P channel expression across the nervous system.

### 2.2 K2P channels expression in rodent

#### 2.2.1 Expression at the peripheral level in rodent

##### 2.2.1.1 Expression in rodent DRG

In DRG, early work in rats established the following hierarchy of K2P expression: **TRESK**, **TRAAK**, **TREK2**, **TWIK2**, **TREK1**, **THIK2**, **TASK1**, **TASK2**, **THIK1**, and **TASK3** (Marsh et al., 2012). In mice, multiple K2P members—including **TASK2**, **TREK1**, **TREK2**, **TRAAK**, **THIK1**, and **TRESK**—are likewise present in DRG neurons (Aller and Wisden, 2008). More recent transcriptomic analyses have refined these observations, ranking K2P expression in mouse DRG from highest to lowest as **THIK2**, **TREK1**, **TASK1**, **TWIK1**, **THIK1**, **TRESK**, **TRAAK**, **TREK2**, and **TWIK3** (Zeisel et al., 2018).

At the cellular level within the DRG, the **TRAAK** displays a relatively uniform expression across different sensory neuron subtypes (Pollema-Mays et al., 2013; Acosta et al., 2014). In contrast, **TREK1** and **TREK2** show a more restricted expression pattern, being enriched in small and medium nociceptive C fibers (Marsh et al., 2012; Yamamoto et al., 2009; Viatchenko-Karpinski et al., 2018). Transcriptomic data corroborate these experimental findings, revealing that **TREK1** is predominantly expressed in peptidergic C fibers, whereas **TREK2** is mainly enriched in non-peptidergic, IB4 (isolectin B4)-positive neurons (Messina et al., 2022). **TREK/TRAAK** channels have also shown to be widely expressed in DRG innervating the mouse colon (La and Gebhart, 2011). **TRESK** is broadly expressed in DRG neurons (Marsh et al., 2012; Lafrenière et al., 2010), including both CGRP (calcitonin gene-related peptide)-positive and IB4-positive populations, and is also present in peripheral terminals innervating the glabrous skin (Liu et al., 2021; Tulleuda et al., 2011). More recently, **TRESK** expression has been detected in MrgprA3 (mas-related G protein-coupled receptor A3)-positive pruriceptive neurons (Llimós-Aubach et al., 2025). **THIK1** and **THIK2** expression is restricted to subsets of small-diameter C-fiber neurons (Zeisel et al., 2018; Haskins et al., 2017), and recent *in situ* hybridization studies indicate that both channels are exclusively expressed in non-peptidergic nociceptors (Gilbert et al., 2026). **TWIK1** is consistently reported as abundant K2P channels in rodent DRG, with expression spanning small, medium, and large neurons (Marsh et al., 2012; Mao et al., 2017). Its cell-type specificity has been clarified through immunohistochemical analyses, revealing localization to large NF200 (neurofilament 200)-positive, TRPV1 (transient receptor potential vanilloid 1)- and IB4-negative neurons (Pollema-Mays et al., 2013). In rats, **TASK1** and **TASK3** are expressed in small nociceptive C fibers but exhibit distinct molecular signatures: **TASK1** is restricted to TRPV1-positive peptidergic neurons, whereas **TASK3** is found in TRPV1/IB4-negative neurons and in TRPM8-positive cold-sensitive neurons (Pollema-Mays et al., 2013; Morenilla-Palao et al., 2014). Interestingly, 95% of **TASK3**-expressing neurons express also **TASK1** subunit, suggesting the possible formation of **TASK3/TASK1** heterodimers in those neurons (Morenilla-Palao et al., 2014; Liao et al., 2019). In mice, transcriptomic analyses confirm the preferential enrichment of **TASK1** in peptidergic neurons (Zeisel et al., 2018). **TALK1** and **TASK2** show only low-level expression in rodent DRG (Marsh et al., 2012; Wang et al., 2019).

Beyond neurons, DRG glial cells also express several K2P channels. Transcriptomic data from the Linnarsson’s group indicate that **TWIK2**, **THIK2**, and **THIK1** are the most abundant K2P members in IBA1 (ionized calcium-binding adapter molecule 1)-positive cells within the mouse DRG (Zeisel et al., 2018). These observations emphasize the involvement of K2P channels not only in neuronal pain transmission but also in glial contributions to inflammatory pain signaling (Luo et al., 2021).

**FIGURE 2:**
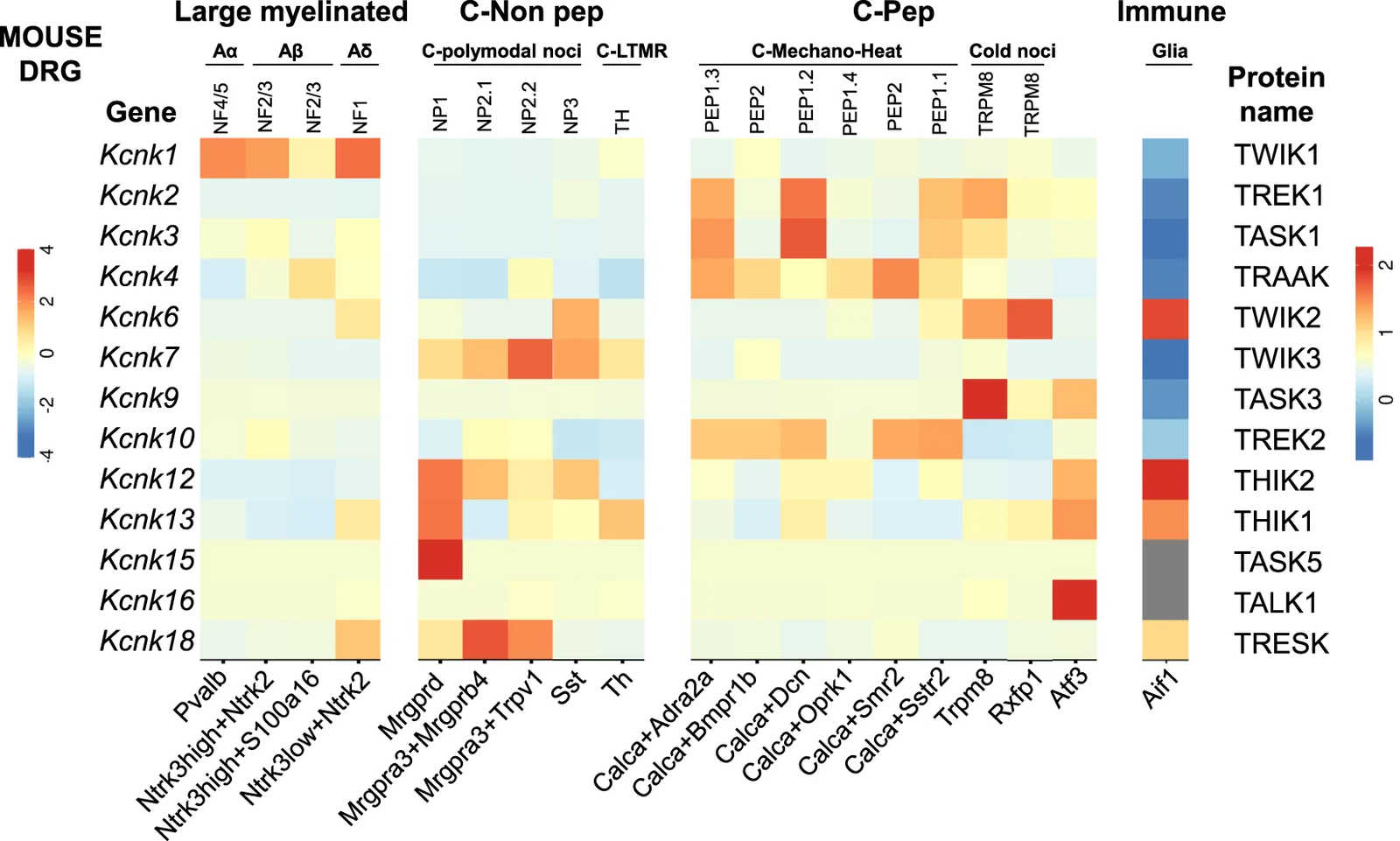
Heatmap showing the expression of K2P channels across different subtypes of mouse sensory DRG neurons and microglia-like immune cells. The heatmap was generated from painseq.shinyapps made by Bhuiyan et al. (2024). The expression levels of K2P channels (Kcnk genes) are shown in mouse dorsal root ganglia (DRG) neuron subpopulations and glial cells. Expression values are color-coded, with red indicating high expression and blue indicating low expression. K2P channel genes (protein): Kcnk1 (TWIK1), Kcnk2 (TREK1), Kcnk3 (TASK1), Kcnk4 (TRAAK), Kcnk6 (TWIK2), Kcnk7 (TWIK3), Kcnk9 (TASK3), Kcnk10 (TREK2), Kcnk12 (THIK2), Kcnk13 (THIK1), Kcnk15 (TASK5), Kcnk16 (TALK1), Kcnk18 (TRESK). Neuron subtypes: NF4/5, NF2/3, NF1: Neurofilament-positive, myelinated neurons (large-diameter), NP1, NP2.1, NP2.2, NP3: Non-peptidergic small-diameter nociceptive neurons, TH: Tyrosine hydroxylase-expressing neurons, PEP1.1-PEP2: Peptidergic small-diameter nociceptive neurons, TRPM8: TRPM8-expressing cold-sensing neurons. Noci stands for nociceptor. Markers: Pvalb: Parvalbumin, Ntrk: Neurotrophic receptor tyrosine kinase, Mrgprd/a3/b4: MAS-related G protein-coupled receptor member D/A3/B4, Trpv1: Transient receptor potential vanilloid 1, Sst: Somatostatin, Th: Tyrosine hydroxylase, Calca: Calcitonin-related polypeptide alpha (encodes CGRPα), Adra2a: Alpha-2A adrenergic receptor, Bmpr1b: Bone morphogenetic protein receptor type 1B, Dcn: Decorin, Oprk1: Opioid receptor kappa 1 (κ-opioid receptor), Smr2: submaxillary gland androgen regulated protein 2, Sstr2: Somatostatin receptor 2; Trpm8: Transient receptor potential melastatin 8, Rxfp1: Relaxin family peptide receptor 1, Atf3: Activating transcription factor 3, Aif1: Allograft inflammatory factor 1 (encode Iba1: ionized calcium-binding adapter molecule 1) which indicates microglia-like immune cells expression.

The most comprehensive evidence to date comes from the meta-analysis of 15 independent mouse RNA-seq datasets performed by Bhuiyan et al. (2024). This large-scale integration confirms robust expression of multiple K2P subunits in mouse DRG, with clear cell-type–specific patterns (Figure 2). Among small nociceptive neurons, **TWIK3**, **THIK2**, **THIK1**, **TASK5**, and **TRESK** are preferentially enriched in non-peptidergic C-fibers, whereas **TREK1**, **TASK1, TRAAK, TWIK2, TASK3** and **TREK2** are more characteristic of peptidergic nociceptors. **TWIK1** exhibits particularly strong expression in larger myelinated neurons. In IBA1/Aif1-positive peripheral immune cells, **TWIK2**, **THIK2**, and **THIK1** emerge as the most abundant K2P subunits, in strong agreement with immunohistochemical evidence from IBA1-positive central microglia (Chen et al., 2014; Marsh et al., 2012; Bhuiyan et al., 2024; Royal et al., 2019).

##### 2.2.1.2 Expression in rodent TG

In the mouse TG, *in situ* hybridization studies indicate that **TRESK** is expressed in roughly half of all neurons (∼51%) (Weir et al., 2019). Size-distribution analyses further show robust **TRESK** expression in Aδ CGRP-positive nociceptive neurons, a pattern that may underlie its involvement in trigeminal pain disorders such as migraine and trigeminal neuropathy (Lafrenière et al., 2010; Weir et al., 2019; Guo et al., 2019; Royal et al., 2019). Additional K2P members are also present in the TG: **TREK2** is expressed in non-peptidergic neurons in rat TG and functionally interacts with GABA_B_ receptor signaling to modulate excitability; **THIK1** has been detected in rat TG and **TASK3** expression has been reported in mouse TG (Liao et al., 2019; Chang et al., 2021; Kang et al., 2014; Lengyel et al., 2020).

**FIGURE 3:**
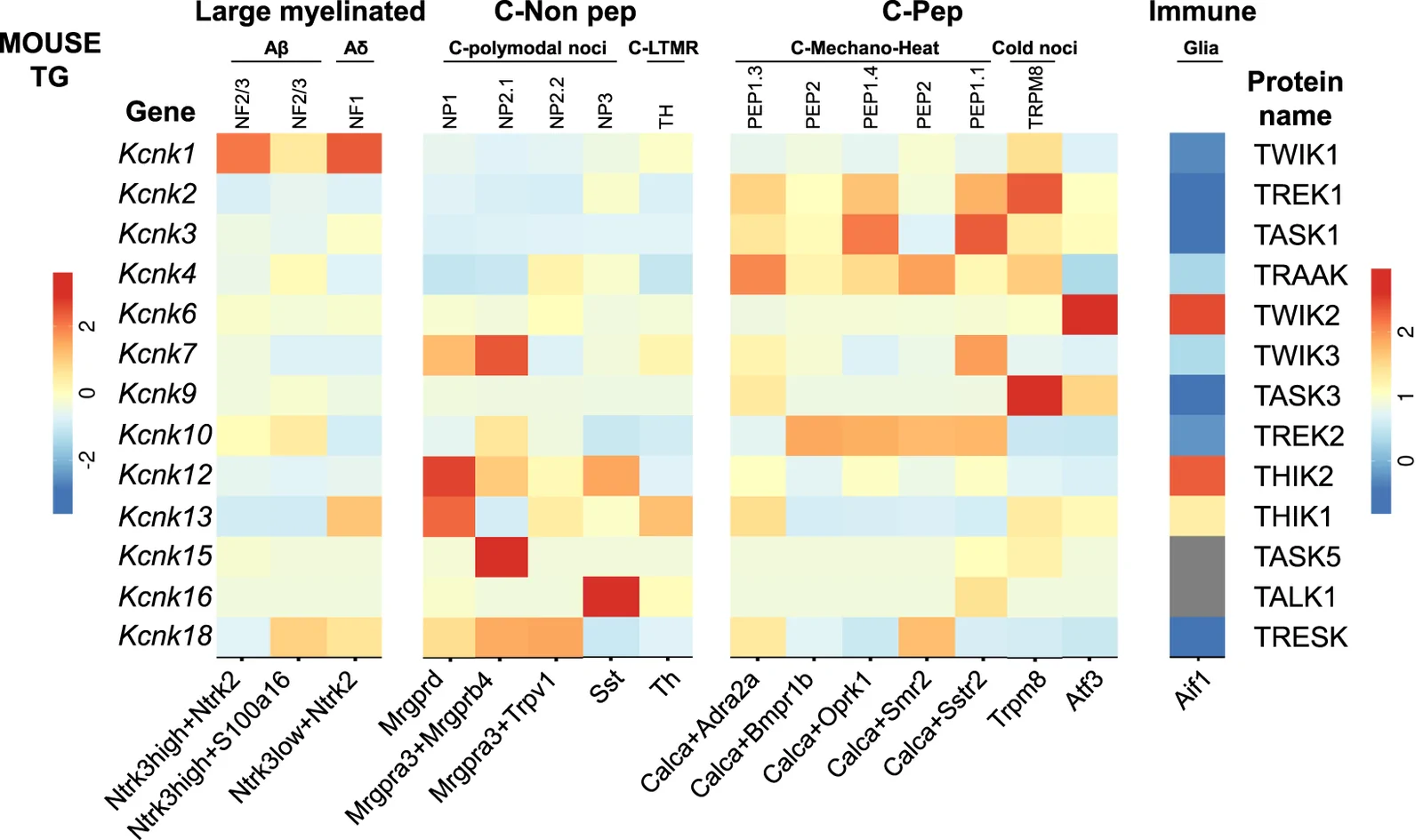
Heatmap showing the expression of K2P channels across different subtypes of mouse sensory TG neurons and microglia-like immune cells. The heatmap was generated from painseq.shinyapps made by Bhuiyan et al. (2024). The expression levels of K2P channels (Kcnk genes) are shown in mouse trigeminal ganglia (TG) neuron subpopulations and glial cells. Expression values are color-coded, with red indicating high expression and blue indicating low expression. K2P channel genes: as in Figure 2. Neurons subtypes: as in Figure 2 + LTMR: Low-threshold mechanoreceptor. Markers: as in Figure 2.

Consistent with findings in DRG, the meta-analysis of 15 independent RNA-seq datasets by Bhuiyan et al. (Bhuiyan et al., 2024) reveals a closely parallel pattern of K2P channel expression in TG neurons (Figure 3). **TWIK3, THIK2**, **THIK1**, **TASK5**, **TALK1**, and **TRESK** are preferentially expressed in non-peptidergic C-fiber nociceptors, whereas **TREK1**, **TASK1**, **TRAAK**, **TWIK2**, **TASK3** and **TREK2** are enriched in C-peptidergic nociceptive populations. **TWIK1** again shows particularly strong expression in larger myelinated A-type neurons. As in DRG, **TWIK2**, **THIK2**, and **THIK1** emerge as the most abundant K2P subunits in IBA1/Aif1-positive peripheral immune cells within the TG (Bhuiyan et al., 2024).

##### 2.2.1.3 Expression at rodent peripheral nerve endings

The peripheral terminals of sensory neurons in the skin and within visceral and other internal sensory organs constitute critical sites for the detection and transmission of acute pain signals (Dubin and Patapoutian, 2010; Westlund, 2000). Changes in the excitability of these peripheral endings profoundly affect the initiation and propagation of nociceptive activity toward the CNS.

In rodents, **TREK1** and **TRAAK**, together with **THIK1**, constitute the principal potassium channels localized at nodes of Ranvier (Escobedo and Rasband, 2025; Kanda et al., 2019; Kanda et al., 2023; Ronzano et al., 2021; Wu et al., 2024). In vagal afferent neurons of the rat nodose ganglia—which innervate visceral organs—**TREK1**, **TRAAK**, **TWIK2**, **TASK1**, and **TASK2** are all detected (Zhao et al., 2010). **TRESK** is present in peripheral nerve terminals innervating the glabrous skin in rats and shows robust expression in mouse D-hair mechanoreceptors (Liu et al., 2021; Tulleuda et al., 2011; Weir et al., 2019).

#### 2.2.2 Expression at the central level in rodent

K2P channel transcripts are widely distributed throughout the CNS, encompassing the cerebrum, cerebellum, brainstem, and spinal cord (Medhurst et al., 2001; Talley et al., 2001). They are also present in supraspinal regions involved in pain modulation—including the cortex, hippocampus, and other integrative centers—where they regulate neuronal excitability and thereby shape pain processing (Luo et al., 2021; García et al., 2019).

##### 2.2.2.1 Expression in rodent spinal cord

K2P channels are broadly expressed in rodent spinal dorsal horn neurons involved in pain processing. In the mouse spinal cord, **TASK1**, **TASK3**, **TREK2**, **TRAAK**, and **TWIK1** show prominent expression (Talley et al., 2001). More recent transcriptomic analyses refine this profile, revealing high expression of **TREK1**, **TWIK1**, and **TASK1**, with much lower levels of **THIK2**, **TREK2**, **THIK1**, and **TRAAK** (Zeisel et al., 2018). **TRESK** is present in the rat and mouse dorsal horns (Hwang et al., 2015; Yoo et al., 2009). In rats, additional K2P members are found in the spinal cord, including **TREK1** and **TASK3**, the latter showing particularly robust expression (García et al., 2019; Blankenship et al., 2013; Hervieu et al., 2001). Developmental studies in rats further indicate that **TWIK1** and **TWIK2** maintain stable postnatal expression, with **TWIK1** consistently more abundant (Blankenship et al., 2013).

##### 2.2.2.2 Expression in rodent supraspinal pain-modulating regions

Several K2P channels are expressed in supraspinal regions although with distinct regional distributions. **TREK1** and **TRAAK** are widely detected, with **TREK1** particularly enriched in the striatum, cortex, and hippocampus, while **TRAAK** shows its highest expression in the cortex. In contrast, **TREK2** exhibits a more restricted pattern, being largely confined to the granule cell layer of the cerebellum (Talley et al., 2001; Hervieu et al., 2001; Gu et al., 2002). **TASK** channels are likewise broadly distributed across the CNS, with **TASK1** predominantly expressed in the cerebellum, whereas **TASK3** is more abundant in the hippocampus, cortex, cerebellum, and in selected nuclei, including the paraventricular nucleus of the thalamus, locus coeruleus, and dorsal raphe (Medhurst et al., 2001; Talley et al., 2001; Rusznak et al., 2004). **TASK1, TASK3, THIK1, THIK2, TREK1,** and **TRAAK** are also highly expressed in the hypothalamus and thalamus, whereas **TWIK1** is predominantly detected in the thalamus. In addition, some of these channels show substantial expression in the amygdala like the **THIK**, the **TASK** and the **TREK** channels (Talley et al., 2001). In addition, high levels of **TRESK** expression have been reported in several supraspinal regions, notably the cortex and the periaqueductal gray (PAG), further supporting a role for K_2P_ channels in central neuronal excitability (Yoo et al., 2009).

##### 2.2.2.3 Expression in rodent non neuronal cells of the central nervous system

In most studies examining K2P channel expression in the CNS, these channels are implicitly considered neuronal. However, several K2P family members are also expressed in non-neuronal CNS cell types, particularly within the glial compartment. **TREK1** and **TWIK1**, for instance, are present in cortical and hippocampal astrocytes, where they might contribute to K^+^ buffering and the regulation of neuronal excitability (Chu et al., 2010; Mi Hwang et al., 2014; Wang et al., 2013; Woo et al., 2012; Zhou et al., 2009). **TWIK1** and **TASK1** are also detected in oligodendrocytes, although their functions in these cells remain unclear (Zeisel et al., 2018; Albrecht et al., 2019; Hawkins and Butt, 2013; Saunders et al., 2018). **THIK1** is predominantly expressed in microglia and has been shown to provide the major K2P conductance, contributing to hippocampal microglial RMP and ramified morphology (Kim et al., 2026; Madry et al., 2018; Drinkall et al., 2022; Rifat et al., 2024; Izquierdo et al., 2021). **TREK1** and **TWIK2** are likewise present in IBA1-positive microglial and macrophage populations, though their functional roles are less well defined (Di et al., 2018; Yan et al., 2022; Huang et al., 2023; Immanuel et al., 2024).

### 2.3 K2P channel expression in human

#### 2.3.1 Expression in periphery in human

##### 2.3.1.1 Expression in human DRG

A human transcriptomic study published in 2015 reported on the expression of K2P channels in DRGs, TG, and various other tissues, and compared these profiles with those observed in mice (Flegel et al., 2015). Nearly all K2P transcripts were detected in human DRG, with the exception of **TALK1**. Among these, **THIK2** displayed the highest mean expression level, whereas **TWIK1** was the most abundant K2P channel in mouse DRG. **TWIK1** and **TASK1** also showed substantial expression in human DRG (Flegel et al., 2015).

**FIGURE 4:**
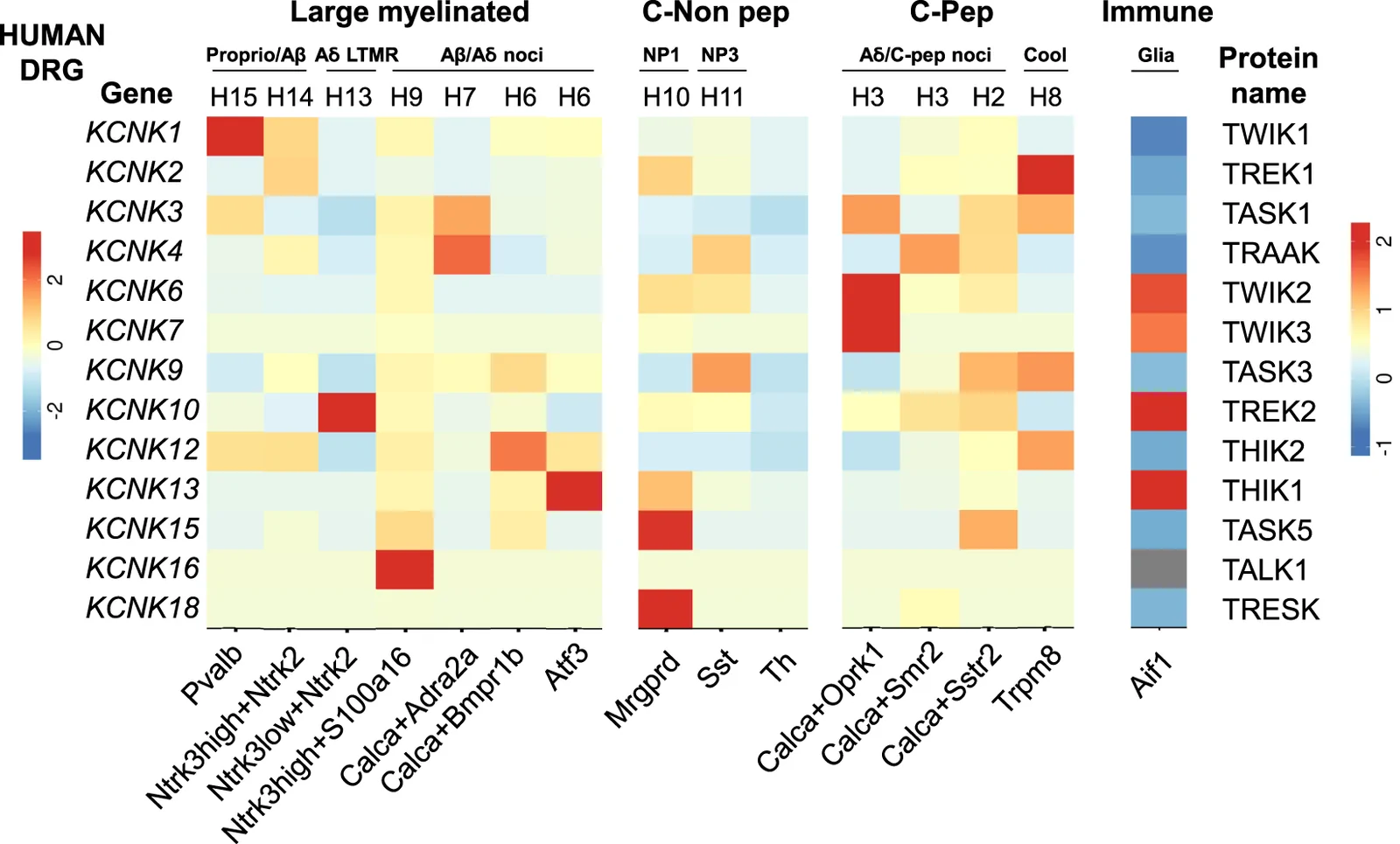
Heatmap showing the expression of K2P channels across different subtypes of Human sensory DRG neurons and microglial-like immune cells. The heatmap was generated from painseq.shinyapps made by Bhuiyan et al. (2024). The expression levels of K2P channels (KCNK genes) are shown in Human dorsal root ganglia (DRG) neuronal subtypes (H1-H15) and glial cells. Expression values are color-coded, with red indicating high expression and blue indicating low expression. K2P channel genes: as in Figure 2. Neurons subtypes: H1–H15 denotes the transcriptomic classification of human DRG sensory neuron subtypes described by Nguyen et al. (2021). These clusters were defined by single-nucleus RNA sequencing and assigned putative functional identities based on their gene expression profiles. Proprio: proprioceptors, LTMR: Low-threshold mechanoreceptor, noci: nociceptor, NP1/NP3: Non-peptidergic small-diameter nociceptive neurons, pep noci: peptidergic nociceptor. Markers: as in Figure 2.

The meta-analysis by Bhuiyan et al. (2024), which integrates three independent human single-cell RNA-seq datasets shows that human DRG neurons exhibit a K2P channel expression profile similar to that observed in mice, with marked cell-type specificity (Figure 4) (Jung et al., 2023; Nguyen et al., 2021; Tavares-Ferreira et al., 2022). **TWIK1** is strongly expressed in large myelinated Aβ non nociceptive neurons which in human are commonly associated with proprioceptors, whereas **TREK2** is enriched in Aδ-LTMRs (low-threshold mechanoreceptors). **TASK1, TRAAK, THIK2, THIK1** and **TALK1** are predominantly expressed in human sensory neuron populations corresponding to myelinated Aβ sensory neurons and nociceptive Aδ neurons. **TASK5** and **TRESK** are enriched in non-peptidergic neurons, while **TREK1, TWIK2, TWIK3** and **TASK1** are expressed in Aδ and C peptidergic nociceptive neurons. Notably, IBA1-positive glial cells display high expression levels of **TWIK2, TWIK3, TREK2** and **THIK1**, closely recapitulating the expression patterns observed in mice (Bhuiyan et al., 2024).

##### 2.3.1.2 Expression in human TG

In the human TG, **TRESK** stands out as a prominently expressed K2P channel and is strongly implicated in migraine with aura, with multiple genetic studies linking LOF variants to increased susceptibility (Lafrenière et al., 2010; Royal et al., 2019; Andres-Bilbe et al., 2020). Beyond **TRESK**, transcriptomic analyses reveal that all K2P genes are expressed in human TG, with **THIK2** representing the most abundant subunit, followed by **TWIK1**, **TREK1**, **TRAAK**, **TASK1**, **TASK2**, and **TRESK**—a distribution that closely mirrors the expression pattern reported in mouse TG (Flegel et al., 2015).

**FIGURE 5:**
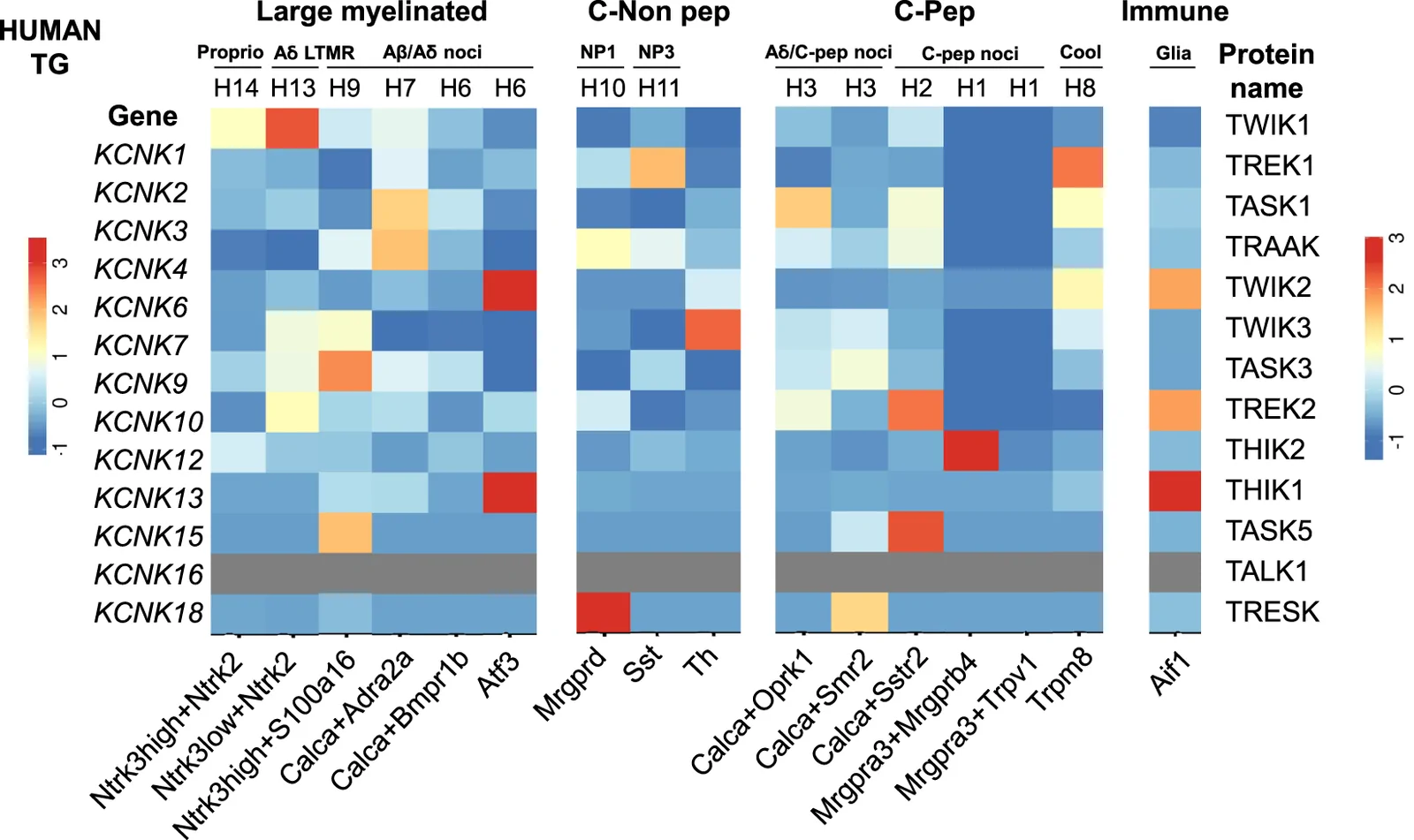
Heatmap showing the expression of K2P channels across different subtypes of Human sensory TG neurons and microglial-like immune cells. The heatmap was generated from painseq.shinyapps made by Bhuiyan et al. (2024). The expression levels of K2P channels (KCNK genes) are shown in Human trigeminal ganglia (TG) neuronal subtypes (H1-H15) and glial cells. Expression values are color-coded, with red indicating high expression and blue indicating low expression. K2P channel genes: as in Figure 2. Neurons subtypes: as in Figure 4. Markers: as in Figure 2.

The recent meta-analysis by Bhuiyan et al. (2024) has provided a detailed, cell-type–resolved map of K2P channel expression in human TG, revealing striking functional specialization (Figure 5). **TWIK1** is still predominantly expressed in large proprioceptive neurons, while **TWIK2**, **TASK3**, and **THIK1** are enriched in large Aβ/Aδ nociceptors. **TWIK3** and **TRESK** show preferential expression in non-peptidergic neurons, whereas **TREK1**, **TREK2**, **THIK2**, and **TASK5** are characteristic of peptidergic nociceptive populations. Notably, **TREK1** displays selective enrichment in cold-sensitive peptidergic nociceptors. Finally, **TWIK3** marks C-LTMRs neurons, a rare and still poorly defined neuronal subset in humans (Nguyen et al., 2021). Notably, **TWIK3** has not yet been validated as a functional K2P channel, as its expression in heterologous systems fails to produce measurable currents, leaving its physiological role unresolved (Salinas et al., 1999).

##### 2.3.1.3 Expression in human peripheral tissues

The RNA-seq dataset generated by Flegel et al. (2015) also provides a broader view of K2P channel expression across human peripheral organs implicated in visceral pain signaling. Several K2P members—including **TWIK1**, **TASK1**, **TASK2**, **TWIK2**, and **TALK2**—show high expression levels in the lungs, suggesting that they could participate in respiratory mechanotransduction or inflammation-mediated sensory pathways. In the colon, **TASK1**, **TASK2**, and **TWIK2** emerge as the predominant subunits possibly supporting visceral nociception (Flegel et al., 2015). Beyond visceral tissues, multiple K2P channels—**TREK1**, **TREK2**, **TRAAK**, **TASK1**, **TASK2**, and **TASK3**—have been detected in the human keratinocyte-derived HaCaT cell line, supporting potential involvement in cutaneous mechanosensation and peripheral inflammatory responses (Kang et al., 2007).

#### 2.3.2 Expression in the human central nervous system

**FIGURE 6:**
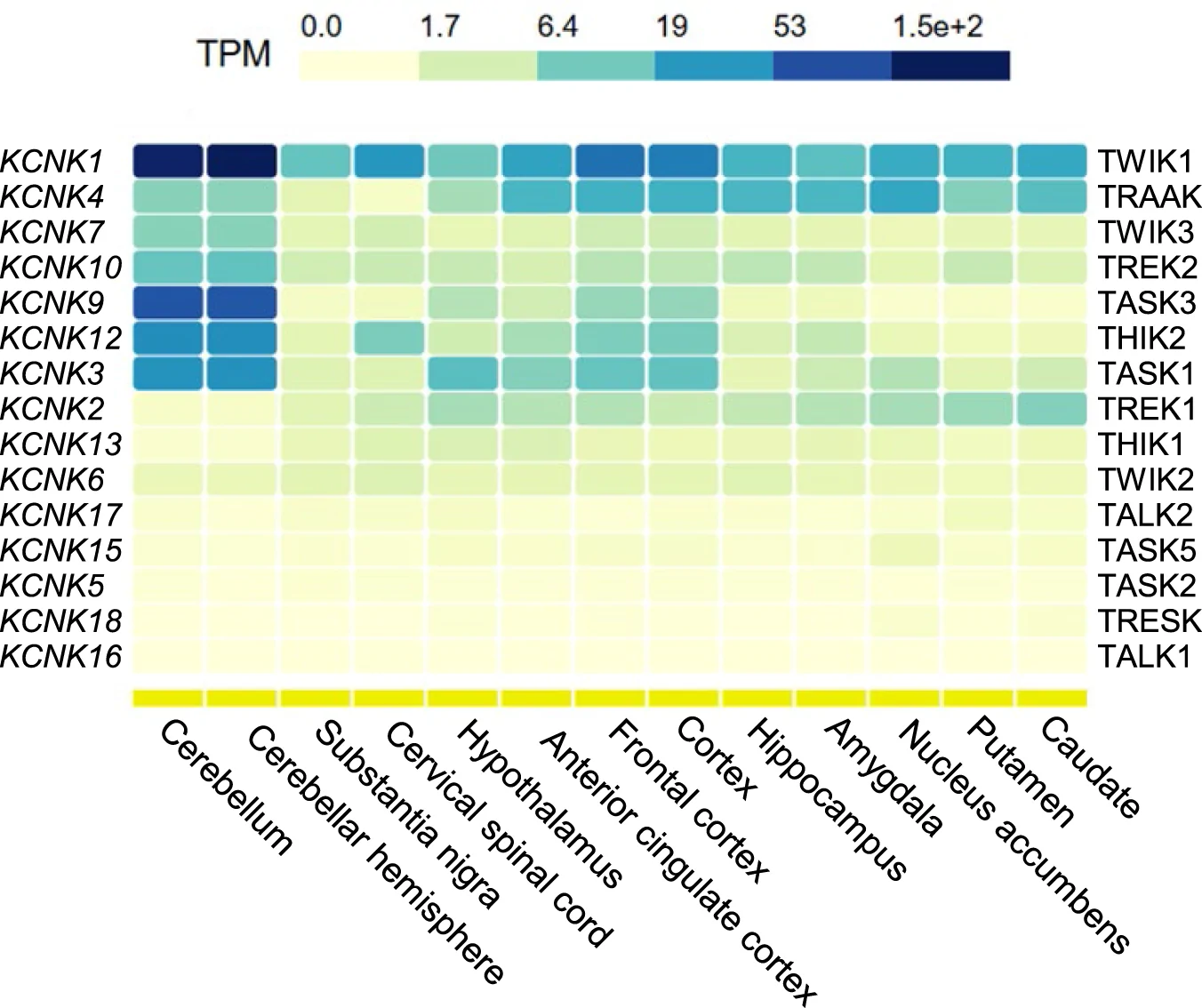
Heatmap showing the expression of K2P channel across the human CNS. Gene expression levels are represented as transcripts per million (TPM) and displayed according to the color scale, ranging from low (light yellow) to high expression (dark blue). K2P gene names are indicated on the left side while the corresponding protein names are shown on the right. Data were retrieved from the Adult Genotype-Tissue Expression (GTEx) Project).

Only limited data are currently available regarding the expression of K2P channels in human brain structures. **TASK1** and **TASK3** have been detected in the human brain, with particularly high expression levels observed in astrocytes of the cerebellum and hippocampus, as well as in Purkinje neurons and granule cells (Rusznak et al., 2004). Strong expression of **THIK2** and **TWIK1** has also been reported in the RNA-seq analysis of human TG and DRG (Flegel et al., 2015). In addition, **THIK1** is highly and functionally expressed in human brain microglia (Drinkall et al., 2022; Rifat et al., 2024). According to GTEx (genotype-tissue expression) data (a major public resource for the analysis using bulk RNA sequencing of human gene expression across tissues), **TWIK1** is the most highly expressed K2P channel in the human CNS (Figure 6). Particularly strong expression was observed in the cerebellum and cerebellar hemisphere, although **TWIK1** transcripts were also detected across most brain regions. These included structures critically involved in pain processing, such as the spinal cord, anterior cingulate cortex (ACC), and amygdala, as well as regions thought to exert more indirect or modulatory influences on nociception, including the cerebellum and basal ganglia. **TRAAK** appears to be the second most highly expressed K2P channel, although its expression in the spinal cord seems comparatively low. Other detectable K2P channels include **TWIK3**, **TREK2**, **TASK3**, **THIK2**, **TASK1**, and **TREK1**. In contrast, **TWIK2**, **TALK2**, **TASK5**, **TASK2**, and **TALK1** displayed relatively low expression levels. **TRESK** expression was not detected in the GTEx dataset, despite the fact that this channel was originally cloned from human spinal cord tissue (Sano et al., 2003) (Figure 6). This may be due to the fact that only cervical spinal cord samples were analyzed, but it may also reflect technical limitations related to bulk RNA-seq sensitivity, tissue sampling, low-abundance cell-specific expression, or differences between healthy donor tissue and experimentally enriched preparations.

### 2.4 Comparison of K2P channel expressions in rodents and humans

Rodent models have been instrumental in establishing the role of K2P channels in nociceptor excitability and pain. However, successful clinical translation requires confirmation that these channels are similarly expressed and functionally relevant in humans. Recent single-cell transcriptomic studies have shown that the overall repertoire of K2P channels is largely conserved between rodents and humans, while revealing important species-specific differences in channel abundance and neuronal subtype distribution (Jung et al., 2023; Nguyen et al., 2021; Tavares-Ferreira et al., 2022; Ray et al., 2018; Yu et al., 2024). Although most available human datasets are still based on relatively small numbers of donors and predominantly on tissues without well-characterized chronic pain conditions, they provide an essential framework for evaluating the translational relevance of rodent findings.

Comparative analyses support several K2P channels as promising therapeutic targets. **TRESK** has the strongest clinical validation through its genetic association with migraine, whereas **TREK1** and **TRAAK** combine conserved expression with robust functional evidence from rodent pain models (Lafrenière et al., 2010; Llimós-Aubach et al., 2025; Royal et al., 2019). **THIK2** is particularly intriguing because it is one of the most abundant K2P channels in human sensory ganglia despite limited functional characterization (Gilbert et al., 2026). **TASK1** and **TASK3** remain promising candidates owing to their conserved expression in human nociceptors, while **THIK1** and **TWIK2** may contribute indirectly to pain through neuroimmune mechanisms. Finally, several K2P channels are expressed in large myelinated sensory neurons, suggesting functions extending beyond classical nociception. Consistent with this notion, a recent study demonstrated that **TWIK1** primarily regulates mechanosensory processing rather than mechanical nociception, supporting the idea that K2P channels contribute to multiple somatosensory modalities (Yang et al., 2026). Overall, these findings highlight that target prioritization should integrate comparative human transcriptomics with functional studies, human genetics, and pharmacological tractability to identify the K2P channels with the greatest potential for clinical translation.

## 3 Regulation of K2P channel expression in pain models

### 3.1 Pain conditions and preclinical models

Pain conditions are classified into acute and chronic categories (Bennett et al., 2019; Nicholas et al., 2019). Chronic pain encompasses inflammatory pain (characterized by tissue injury and immune cell infiltration), neuropathic pain (resulting from PNS or CNS injury), and nociplastic pain (centralized pain without clear peripheral pathology) (Xu and Yaksh, 2011). In murine models, most K2P channel studies focus on chronic inflammatory and neuropathic pain paradigms.

**FIGURE 7:**
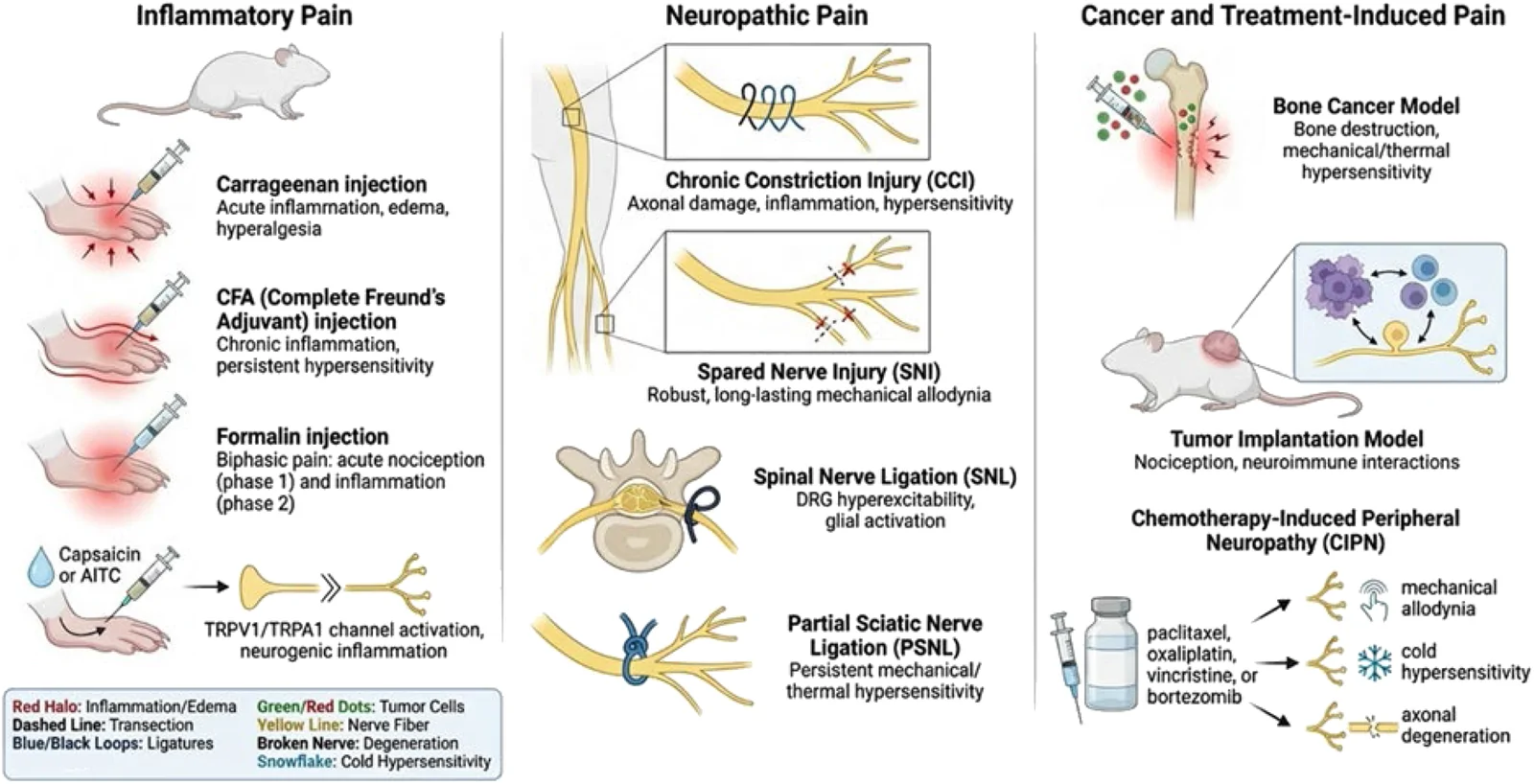
Common preclinical rodent models used to investigate pain mechanisms. Representative experimental models of inflammatory pain, neuropathic pain, and cancer- and treatment-induced pain. Inflammatory pain models include carrageenan, complete Freund’s adjuvant (CFA), and formalin injections, as well as capsaicin or allyl isothiocyanate (AITC) administration to activate TRPV1/TRPA1 channels. Neuropathic pain models include chronic constriction injury (CCI), spared nerve injury (SNI), spinal nerve ligation (SNL), and partial sciatic nerve ligation (PSNL). Cancer-related pain models comprise bone cancer and tumor implantation models, whereas chemotherapy-induced peripheral neuropathy (CIPN) is induced by anticancer agents such as paclitaxel, oxaliplatin, vincristine, or bortezomib. These models reproduce distinct pathological features of pain, including inflammation, peripheral nerve injury, neuroimmune interactions, axonal degeneration, and mechanical, thermal, or cold hypersensitivity.

In preclinical studies in mice and rats, several models are used to reproduce the mechanisms underlying inflammatory pain by inducing local tissue inflammation and nociceptor sensitization (Figure 7, left panel) (Gregory et al., 2013; Mogil, 2009). Commonly used models include carrageenan injection, which produces acute inflammation with edema and mechanical and thermal hyperalgesia; Complete Freund’s Adjuvant (CFA), which induces a long-lasting inflammatory response and persistent hypersensitivity and the formalin test, characterized by a biphasic nociceptive response reflecting both direct nociceptor activation and subsequent inflammatory sensitization. Other models rely on the injection of capsaicin or allyl isothiocyanate (AITC), which activate TRPV1 or TRPA1 (transient receptor potential ankyrin 1) channels and trigger neurogenic inflammation through the release of neuropeptides (Bautista et al., 2006; Caterina et al., 1997; Tjølsen et al., 1992; Winter et al., 1962). Together, these models reproduce key features of inflammatory pain and are widely used to investigate nociceptive mechanisms and evaluate potential analgesic therapies.

To reproduce the key mechanisms underlying neuropathic pain, several experimental models have been developed including peripheral nerve injury, sensory neuron hyperexcitability, and neuroinflammation (Figure 7, middle panel). Most commonly used models rely on partial lesions of the sciatic nerve. In the Chronic Constriction Injury (CCI) model, several loose ligatures are placed around the sciatic nerve, producing chronic compression that results in axonal damage, inflammation, and both mechanical and thermal hypersensitivity. In the Spared Nerve Injury (SNI) model, two branches of the sciatic nerve—the tibial and common peroneal nerves—are transected, while the sural nerve is left intact. This procedure produces robust and long-lasting mechanical allodynia and is considered one of the most reliable and reproducible models of neuropathic pain. Other commonly used paradigms include the Spinal Nerve Ligation (SNL) model and the Partial Sciatic Nerve Ligation (PSNL). In these models, either a spinal nerve root (typically L5 or L5/L6) is tightly ligated near the vertebral column, or only a portion of the sciatic nerve is ligated. These injuries lead to marked hyperexcitability of DRG neurons, activation of spinal glial cells, and persistent mechanical and thermal hypersensitivity (Mogil, 2009; Bennett and Xie, 1988; Decosterd and Woolf, 2000; Ho Kim and Mo Chung, 1992).

Several preclinical models have also been developed in rodents to study cancer-related pain and treatment-induced neuropathic pain (Figure 7, right panel) (Falk and Dickenson, 2014; Flatters et al., 2017). Cancer pain is most commonly modeled using bone cancer models, in which tumor cells are injected into the femur or tibia, leading to progressive bone destruction, inflammation, and robust mechanical and thermal hypersensitivity that resemble metastatic bone pain in patients (Haroun et al., 2023). Other approaches include tumor implantation models, where cancer cells are injected subcutaneously or into peripheral tissues to study tumor-induced nociception and interactions between cancer cells, immune cells, and sensory neurons (Haroun et al., 2023). In addition, chemotherapy-induced peripheral neuropathy (CIPN) models are widely used to mimic pain caused by anticancer treatments (Gadgil et al., 2019). Repeated administration of drugs such as paclitaxel, oxaliplatin, vincristine or bortezomib induces sensory neuropathy characterized by mechanical allodynia, cold hypersensitivity, and axonal degeneration of peripheral sensory fibers (Holmes et al., 1998).

### 3.2 K2P channel expression in inflammatory pain models

In rat models of inflammatory pain, formalin injection produces a rapid and transient upregulation of **TASK1** and **TASK3** protein levels in the ipsilateral L5 DRG, and increases **TASK3** expression in the dorsal spinal cord. These effects are spatially restricted, as no changes are detected contralaterally or in the ventral spinal cord. **TASK3** levels in the DRG also show a delayed decrease several days after formalin administration (García et al., 2019).

In complete Freud’s adjuvant (CFA) inflammatory pain models in rats, the expression of multiple K2P channels is dynamically regulated within days of inflammation, with significant changes in mRNA levels observed over this time frame. Specifically, there is a decrease in **TRESK** expression in DRG of rats at 4 days post-CFA injection compared to day one, correlating with increased spontaneous pain (spontaneous foot lifting behavior) (Marsh et al., 2012). This downregulation is thought to contribute to inflammation-induced disinhibition of nociceptor firing. Four days post-CFA, both **TASK1** and **TASK3** channels show bilateral decreases in rat DRG expression compared to day one post-injection (Marsh et al., 2012). Notably, inflammatory pain states are associated with bilateral downregulation of **TASK1** and **TASK3** expression in DRG neurons, correlating with increased spontaneous pain and supporting a causal role for reduced **TASK**-mediated leak conductance in inflammatory pain pathogenesis (Tulleuda et al., 2011). A decrease in total DRG tissue expression and pixel density of **TASK2** protein has also been observed at day four post-CFA injection compared to day one (Marsh et al., 2012). This downregulation of pH-sensitive K2P channels may account for the acidic environment created by inflammation. **THIK2** channel expression is also reduced at day four post-CFA compared with day one. In contrast, the only K2P channel upregulated under these conditions is **THIK1** (Marsh et al., 2012). Indeed, **THIK1** expression is increased at day four post-CFA, which may suggest a less anti-nociceptive role compared with other K2P channels. Except for **THIK1**, this strong and replicable decrease in K2P channels expression observed post-inflammation in rats is consistent with an increase in the potential excitability of DRG neurons, and thus with enhanced inflammatory pain, as reflected by the increased incidence of spontaneous foot lifting (Marsh et al., 2012). Interestingly, experimental colitis induced by TNBS (2,4,6-trinitrobenzene sulfonic acid) leads to a downregulation of **TREK1** channel expression and impaired mechanosensitivity of **TREK2** channels (La and Gebhart, 2011).

### 3.3 K2P channel expression in neuropathic pain models

In chronic constriction injury (CCI), **TALK1** is downregulated in DRG neurons via inflammatory mechanisms involving S100A9, contributing to nociceptive hypersensitivity (Liu et al., 2025). **TWIK1** expression is likewise reduced in the spinal cord through microRNA-dependent pathways (Xie et al., 2023). More recently, **TWIK1** expression has been reported to be reduced in a CCI model applied to rat. This decrease is modulated by dynamic regulation of angiotensin-II type 1 and type 2 receptors (AT1R and ATR2) suggesting a potential link between the renin-angiotensin system and **TWIK1** in the regulation of sensory neuron excitability in pathological pain (Peralta et al., 2026). Conversely, **TREK1** expression may increase at later chronic stages, with microRNA-mediated modulation influencing pain sensitivity (Shi et al., 2018).

In spinal nerve ligation (SNL), several K2P channels undergo distinct regulatory changes in primary sensory neurons. **TWIK1** expression is selectively downregulated in ipsilateral DRGs (Mao et al., 2017). Nerve injury downregulated **TREK1** mRNA in injured L5 DRG and uninjured L4 DRG during the first 7 days (García et al., 2021a). These mRNA levels returned to baseline levels 14 days after injury. These results agree with those showing that oxaliplatin decreases **TREK1** mRNA in DRG of mice (Descoeur et al., 2011). In contrast, other reports have demonstrated that nerve injury upregulates **TREK1** mRNA in mice and rats (Shi et al., 2018; Han et al., 2016). **TREK1** function is also impaired following SNL: pharmacological activation of the channel reverses tactile allodynia, underscoring its antinociceptive contribution (García et al., 2021a). **TASK3** expression is decreased in DRGs but remains unchanged in the spinal cord (García et al., 2019), whereas **TRESK** exhibits a bidirectional regulation—downregulated in the DRG but upregulated in the dorsal horn (Hwang et al., 2015). In rat models of SNL, **TASK1** expression remains largely unchanged in DRGs and in the spinal cord. In contrast, **TASK3** is significantly downregulated in ipsilateral L4–L5 DRGs from day 1 after injury, with recovery occurring in the uninjured L4 DRG but persisting in the injured L5 DRG. No significant modulation of **TASK**3 is observed in the dorsal or ventral spinal cord (García et al., 2019).

In the spared nerve injury (SNI) model, both **TWIK1** and **TASK3** are downregulated in DRG neurons, whereas **TASK1** is upregulated (Pollema-Mays et al., 2013). A robust and sustained downregulation of **TWIK1** expression occurs in ipsilateral DRG from one to 4 weeks following SNI, with expression remaining significantly reduced at all timepoints (Pollema-Mays et al., 2013). This persistent suppression suggests a role for **TWIK1** in maintaining neuropathic pain states. In the same study, while **TASK3** shows transient downregulation at one-week post-SNI, it reverts to baseline after 2 weeks, suggesting that transient **TASK3** suppression may be important for establishing neuropathic pain, but not for its maintenance. **TRESK** expression decreases in both DRG and spinal cord, and its experimental overexpression attenuates mechanical allodynia and dampens MAPK (mitogen-activated protein kinase)-dependent inflammatory signaling (Zhou et al., 2013).

Following spinal nerve axotomy (SNA), **TREK2** expression in IB4-positive neurons is tightly dependent on GDNF (glial cell line-derived neurotrophic factor) signaling; loss of this trophic support reduces **TREK2** and contributes to neuronal hyperexcitability (Messina et al., 2022). **TRESK** is similarly downregulated after axotomy and its reduction correlates with increased sensory neuron firing (Tulleuda et al., 2011). In a sciatic nerve axotomy model, a decrease in **TRESK** expression is observed in the DRG of rats (Tulleuda et al., 2011).

### 3.4 K2P channel expression in migraine and headache models

We found extremely few studies investigating the regulation of K2P channel expression in animal or human models of migraine or headache. The only two studies identified involved the **TWIK1** channel. The first study focused on trigeminal neuralgia, in which human genetic studies implicated **TWIK1** duplication in the disease (Dong et al., 2020). The second study was performed in a rabbit model of trigeminal neuropathy induced by spinal compression, where **TWIK1** mRNA expression was found to be increased in the TG, infraorbital nerve, and dorsal horn, highlighting species- and model-specific regulation (Sarıtaş et al., 2025).

### 3.5 K2P channel expression in cancer-associated pain and cancer-treatment associated pain

Accumulating evidence indicates that K2P channel expression is dynamically modulated in cancer-associated pain and in pain induced by anticancer treatments. In models of cancer-induced bone pain, tumor-derived VEGF (vascular endothelial growth factor) activates VEGFR2 (VEGF receptor 2) signaling in sensory neurons, upregulating the calcineurin inhibitor RCAN1 (regulator of calcineurin 1) and thereby suppressing calcineurin-dependent transcription of **TRESK**, a change that contributes to pain hypersensitivity (Yang et al., 2018). Consistently, reduced **TRESK** mRNA expression has been reported in the DRG of rats with bone metastases, correlating with spontaneous pain, hyperalgesia, and decreased potassium currents in small-diameter neurons (Liu et al., 2021; Yang et al., 2018). Chemotherapy-induced peripheral neuropathies also involve altered K2P channel expression: in paclitaxel-treated mice, **TWIK1** expression is downregulated through DNMT3 (DNA methyltransferase 3)-dependent promoter methylation (Mao et al., 2019). Oxaliplatin-evoked neuropathy has similarly been linked to decreased **TREK1, TREK2** and **TRESK** expression in DRG neurons, an effect reversible by sodium butyrate treatment via epigenetic mechanisms involving modulation of histone acetylation (Ho et al., 2025; Pereira et al., 2021). Supporting a causal role for K2P channel dysfunction in tumor-associated pain, pharmacological activation of **TREK1** with riluzole alleviates mechanical hypersensitivity and spontaneous pain in a tibial bone cancer model (Delanne-Cuménal et al., 2024). Finally, clinical studies also suggest altered K2P channel regulation in cancer-related pain states: breast cancer patients experiencing persistent pain after surgery show variable expression of the **TASK3** gene associated with specific SNPs (single nucleotide polymorphism), although the functional direction of these variants remains to be determined (Zhang et al., 2021).

Although most pain models are associated with a downregulation of K2P channel expression at peripheral level, the direction and magnitude of these changes depend on the channel subtype, neuronal population, anatomical level, and pathological context (Table 1) This complexity underscores the need for cell-type-specific and temporally resolved analyses.

**TABLE 1:** Regulation of K2P channels expressions in experimental pain models. Summary of reported changes in KCNK gene expression and corresponding K2P channel subtypes across different pain models and pathological conditions. The table highlights the direction of K2P channel regulation, the affected tissues within the nociceptive pathway, including DRG and spinal cord, and the associated references.

Gene	Channel	Regulation across pain models	Localization	References
*Kcnk1*	**TWIK1**	↓ CCI, SNL and SNI neuropathic pain Altered expression in trigeminal neuralgia ↑ Trigeminal compression model ↓ Paclitaxel neuropathy	DRG, spinal cord TG	Pollema-Mays et al. (2013), Sarıtaş et al. (2025), Mao et al. (2017), Xie et al. (2023), Dong et al. (2020), Mao et al. (2019)
*Kcnk2*	**TREK1**	↓ Experimental colitis Transient ↓ after SNL then ↑ at chronic stages Impaired after SNL ↓ Oxaliplatin neuropathy	DRG	La and Gebhart (2011), Shi et al. (2018), Han et al. (2016), Pereira et al. (2021), Delanne-Cuménal et al. (2024), García et al. (2021b)
*Kcnk3*	**TASK1**	↑ Early after formalin then ↓ later ↓ CFA inflammatory pain ↔ SNL ↑ SNI	DRG, dorsal spinal cord	Pollema-Mays et al. (2013), Marsh et al. (2012), Tulleuda et al. (2011), García et al. (2019)
*Kcnk5*	**TASK2**	↓ CFA inflammatory pain	DRG	Marsh et al. (2012)
*Kcnk9*	**TASK3**	Transient ↑ after formalin then ↓ ↓ CFA, SNL and SNI Altered expression associated with SNPs	DRG, dorsal spinal cord	Pollema-Mays et al. (2013), Marsh et al. (2012), García et al. (2019), Zhang et al. (2021)
*Kcnk10*	**TREK2**	Impaired function in colitis ↓ Spinal axotomy ↓ Oxaliplatin neuropathy	DRG	Messina et al. (2022), La and Gebhart (2011), Pereira et al. (2021)
*Kcnk12*	**THIK2**	↓ CFA inflammatory pain	DRG	Marsh et al. (2012)
*Kcnk13*	**THIK1**	↑ CFA inflammatory pain	DRG	Marsh et al. (2012), Wu et al. (2024)
*Kcnk16*	**TALK1**	↓ CCI neuropathic pain	DRG	Liu et al. (2025)
*Kcnk18*	**TRESK**	↓ CFA inflammatory pain ↓ DRG but ↑ dorsal horn after SNL ↓ SNI ↓ Spinal axotomy ↓ Cancer-induced bone pain ↓ Oxaliplatin neuropathy	DRG, spinal cord	Marsh et al. (2012), Tulleuda et al. (2011), Hwang et al. (2015), Yang et al. (2018), Ho et al. (2025), Zhou et al. (2017)

Abbreviations: DRG, dorsal root ganglion; TG, trigeminal ganglion; CFA, complete Freund’s adjuvant; CCI, chronic constriction injury; SNL, spinal nerve ligation; SNI, spared nerve injury; SNPs, single nucleotide polymorphisms.

### 3.6 Linking K2P channel expression and function in pain

Overall, K2P channels tend to be downregulated across experimental pain models, consistent with the idea that the loss of hyperpolarizing conductance contributes to sensory neuron hyperexcitability. However, this general trend is accompanied by marked context-dependent regulation. **TRESK** is consistently downregulated following inflammatory and peripheral nerve injury, whereas **TREK1** exhibits heterogeneous regulation (Marsh et al., 2012; La and Gebhart, 2011; Tulleuda et al., 2011; Hwang et al., 2015; Yang et al., 2018; Ho et al., 2025; Pereira et al., 2021; Delanne-Cuménal et al., 2024; García et al., 2021b; Zhou et al., 2017). These discrepancies likely reflect differences in the underlying mechanisms of distinct pain conditions, with **TREK1** upregulation after nerve injury potentially representing a compensatory response to hyperexcitability, whereas its downregulation in metabolic neuropathies may result from altered trafficking or degradation. Species- and strain-dependent differences, temporal evolution after injury, and methodological variability further complicate the interpretation of K2P channel expression across studies.

Transcriptomic datasets provide a snapshot of gene expression but only partially predict protein abundance, and cannot capture subcellular localization, post-translational modifications, membrane trafficking, or channel gating, all of which critically determine the physiological contribution of ion channels (Vogel and Marcotte, 2012). Proteomic approaches provide information that is generally closer to the cellular phenotype although they also have important limitations, particularly for ion channels which remain technically challenging to quantify, and total protein abundance often correlates poorly with physiological function (Aebersold and Mann, 2016). Ultimately, causal evidence for the involvement of K2P channels in pain relies on functional studies combining electrophysiology, pharmacology and genetic GOF or LOF approaches. Consequently, apparent discrepancies between transcriptomic, proteomic and functional studies often reflect different levels of biological regulation rather than true inconsistencies, highlighting that transcriptomic and proteomic are primarily hypothesis-generating tools, whereas functional validation remains essential for establishing channel function and prioritizing therapeutic targets.

## 4 Genetic insights into a role of K2P channels in pain

### 4.1 Ion channels and neuronal excitability

Ion channels play a central role in the regulation of neuronal excitability and, as such, are key determinants of pain-related behaviors (Eglen et al., 1999). In nociceptive neurons, the balance between depolarizing excitatory currents and hyperpolarizing inhibitory currents dictates activation thresholds, firing frequency, and the transmission of pain signals (Waxman and Zamponi, 2014).

**FIGURE 8:**
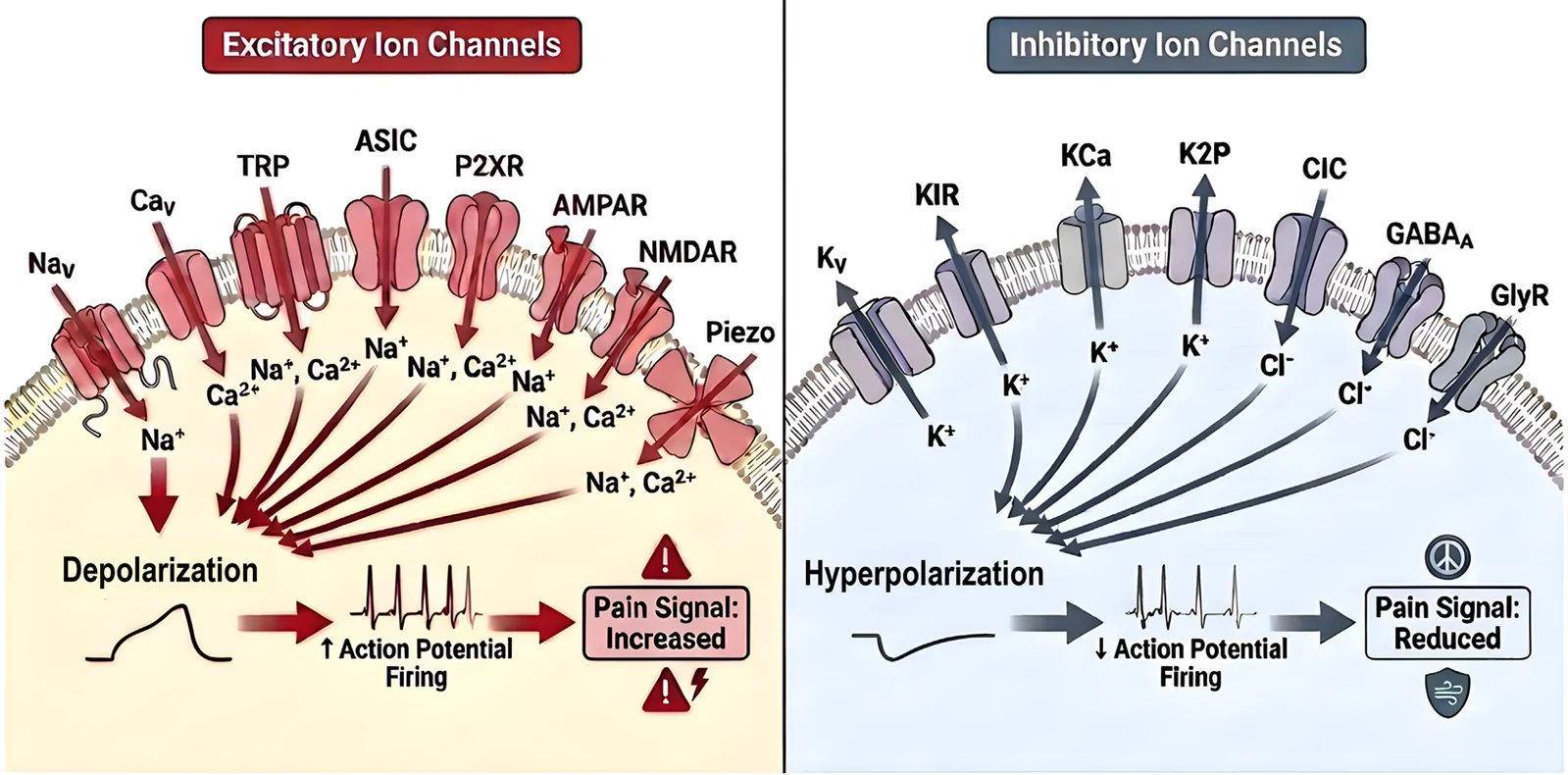
Opposing roles of excitatory and inhibitory ion channels in the regulation of neuronal excitability and pain signaling. In the left panel, excitatory ion channels, including NaV, CaV, TRP channels, ASIC, P2XR, AMPAR and NMDAR glutamate receptors, and mechanosensitive Piezo channels, promote cation influx, membrane depolarization, and action potential generation, thereby enhancing nociceptive transmission. In contrast, the right panel illustrates inhibitory ion channels, mainly potassium and chloride channels, which stabilize the resting membrane potential, promote membrane hyperpolarization, and limit neuronal firing. Among them, K2P channels generate background potassium leak currents that act as key regulators of neuronal excitability and endogenous brakes of pain signaling. Abbreviations: NaV: Voltage-gated sodium channel, CaV: Voltage-gated calcium channel, TRP: Transient receptor potential channel, ASIC: Acid-sensing ion channel, P2XR: purinergic receptor; AMPAR: α-amino-3-hydroxy-5-methyl-4-isoxazolepropionic acid receptor, NMDAR: N-methyl-D-aspartate receptor, KV: Voltage-gated potassium channel; KIR: Inwardly rectifying potassium channel, KCa: Calcium-activated potassium channel, K2P: Two-pore domain potassium channel, ClC: Chloride channel, GABA_A_R: γ-aminobutyric acid type A receptor, GlyR: glycine receptor.

Depolarizing cation channels, including voltage-gated sodium and calcium channels as well as certain non-selective TRP channels, ASIC (acid-sensing ion channel) channels and P2XR (purinergic P2X receptor) receptors promote positive charge influx, membrane depolarization, and action potential initiation; their hyperactivity or overexpression is generally associated with increased nociceptor excitability and pro-nociceptive states, contributing to both acute and chronic pain (Figure 8, left panel). In contrast, hyperpolarizing ion channels, particularly potassium and chloride channels, exert anti-nociceptive effects by stabilizing the membrane potential, increasing resistance to action potential generation, and limiting the spread of excitatory signals (Figure 8, right panel). Reduced activity or dysfunction of these inhibitory channels leads to neuronal disinhibition and pain hypersensitivity. Thus, pain emerges largely from a dynamic imbalance between excitatory and inhibitory ionic mechanisms, making ion channels critical targets for understanding and modulating pathological pain states (Benarroch, 2015; Salzer et al., 2019; Tsantoulas and McMahon, 2014).

K2P channels provide neurons with a constitutive background potassium conductance that acts as a permanent inhibitory brake on electrical excitability by stabilizing the RMP and limiting depolarization. Activation of K2P channels—whether by physiological regulators such as fatty acids, pH changes, mechanical stretch, or temperature, by synthetic pharmacological agents, or through increased channel abundance at the plasma membrane via enhanced trafficking, gene or protein expression, or the expression of GOF mutant channels in animal models or human pathologies—strengthens this inhibitory tone. Such conditions are therefore expected to reduce neuronal excitability and dampen pain signaling, leading to analgesic or hypoalgesia phenotypes. Conversely, stimuli or mechanisms that decrease K2P channel activity, impair their membrane trafficking, reduce their protein expression levels, alter their ion selectivity, or suppress their expression through molecular tools such as small interfering RNA (siRNA), as well as genetic ablation in KO animal models, relieve this inhibitory constraint. The resulting increase in neuronal excitability enhances the firing of pain-transmitting neurons and promotes exaggerated nociceptive behaviors. Using these approaches, numerous studies have demonstrated the involvement of specific K2P channels in pain mechanisms, while further investigations are still required to clearly delineate the processes that are directly controlled by K2P channels from those that fall outside their regulatory scope (Luo et al., 2021; Bista et al., 2015; Ehling et al., 2015; Li and Toyoda, 2015; Ryoo and Park, 2016; Steinberg et al., 2015; Talley et al., 2003; Verkest et al., 2021).

### 4.2 Effects of K2P channels modulation on nociceptor excitability

#### 4.2.1 Effects of decreasing K2P channel expression

Although several K2P channels are expressed in sensory neurons, their contribution to nociceptor excitability differs markedly between family members.

Despite the robust expression of **TWIK1** in DRG neurons, no study had previously demonstrated a functional role for this channel in regulating their electrical activity. This view has recently been refined by Yang et al. (2026), who reported that DRG neurons isolated from mice subjected to CCI are less hyperexcitable in **TWIK1** KO mice than in WT animals. Although these findings suggest that **TWIK1** contributes to the injury-induced hyperexcitability of sensory neurons, they should be interpreted with caution, as the electrophysiological recordings were performed on dissociated cultured DRG neurons, which may not fully preserve the electrical properties acquired *in vivo* after nerve injury. In the same study, the authors further demonstrated that selective deletion of **TWIK1** in spinal inhibitory interneurons (GABAergic interneurons) increases neuronal firing frequency, particularly in response to strong depolarizing current injections (Yang et al., 2026). These findings are consistent with the canonical role of **TWIK1** as a background K^+^ channel contributing to leak conductance and limiting neuronal excitability in these cells, while highlighting the context- and cell type-dependent functions of **TWIK1** within the somatosensory system. Altered excitability has also been reported in non-nociceptive contexts, including dentate gyrus neurons and astrocytes following **TWIK1** knockdown (KD) using short hairpin RNA (shRNA) (Zhou et al., 2009; Yarishkin et al., 2014).

In contrast, modulation of **TREK** and **TRAAK** channel expression has been largely shown to exert a major influence on nociceptor excitability. LOF of **TREK1** lowers heat activation thresholds in C-fibers, increases action potential firing in skin–nerve preparations, and enhances calcium responses in DRG neurons (Alloui et al., 2006). These effects result from membrane depolarization, increased input resistance, and reduced rheobase caused by the loss of **TREK1**-mediated background K^+^ conductance. Similarly, siRNA-mediated KD of **TREK2** depolarizes DRG neurons by approximately 10 mV, and following L5 spinal nerve axotomy, a reduction in membrane-associated **TREK2** in C-fibers is accompanied by comparable depolarization (Acosta et al., 2014). Combined KO of **TREK1** and **TREK2** further amplifies nociceptor hyperexcitability revealing complementary and partially redundant roles of these channels in controlling sensory neuron excitability (Noël et al., 2009). Similarly, expression of a truncated **TRESK** channel having dominant negative effect on **TREK1** and **TREK2** in TG neurons of mice increased neuronal activity (Royal et al., 2019). Consistent with these cellular findings, in skin–saphenous nerve preparations polymodal C-fibers from **TREK1** KO mice display enhanced heat sensitivity across the 30 °C–45 °C range compared with wild type (WT) animals (Alloui et al., 2006). The proportion of C-fibers activated by warming from 30 °C to 50 °C is also significantly increased in **TRAAK** KO mice (Noël et al., 2009). While single deletion of **TREK1** or **TRAAK** does not markedly affect cold sensitivity, combined deletion profoundly alters cold-responsive fiber populations, increasing the proportion of DRG neurons activated at 12 °C from ∼25% to ∼54%. This effect reflects a functional shift in C-fiber response profiles rather than neuronal loss, linking **TREK/TRAAK** activity to modality-specific tuning of nociceptor excitability (Pereira et al., 2014). Beyond nociception, **TREK1** and **TRAAK** also regulate action potential conduction at nodes of Ranvier, highlighting their broader role in axonal excitability and signal propagation (Kanda et al., 2019; Brohawn et al., 2019; Luque-Fernández et al., 2024).

Electrophysiological recordings showed that TRPM8-positive sensory neurons from **TASK3** KO mice are significantly more excitable than WT neurons. **TASK3** deletion induced a ∼5 mV depolarization of the membrane and increased action potential firing in response to depolarizing current injections, without affecting action potential threshold (Morenilla-Palao et al., 2014). These results indicate that **TASK3** normally acts as a negative regulator of sensory neuron excitability, and its loss enhances cold-sensitive neuronal activity. Conditional deletion of **TASK3** in Neuromedin B (NMB)-positive neurons enhances pruritogen-induced scratching and activates gastrin-releasing peptide (GRP)-positive neurons in the spinal dorsal horn (Luo et al., 2026).

Studies using mice with either total or functional KO of **TRESK** consistently demonstrate that loss of this channel enhances the excitability of nociceptive sensory neurons, confirming its key role in controlling neuronal excitability (Andres-Bilbe et al., 2020). In DRG neurons from **TRESK-G339R** functional KO mice, outward K^+^ current is significantly reduced compared to WT littermates (Dobler et al., 2007). Although the RMP is not significantly altered, current-clamp recordings reveal clear changes in action potential waveform, including altered duration and after-hyperpolarization amplitude, together with a marked reduction in rheobase, ultimately leading to increased neuronal excitability (Dobler et al., 2007). Similarly, current-clamp recordings showed that neurons expressing dominant-negative mutant **TRESK** subunits (F139WfsX24) had a higher input resistance, a lower current threshold for action potential initiation, and a higher spike frequency in response to suprathreshold stimuli, indicating that the mutation resulted in hyperexcitability of TG neurons (Pettingill et al., 2019). At the cellular level, **TRESK**-deficient neurons exhibit reduced activation thresholds and increased firing rates, effects that are further amplified under inflammatory conditions (Weir et al., 2019). At the integrated level, this heightened excitability translates into enhanced sensory function, as nociceptive C-fibers from **TRESK** KO mice display increased responses to cold and mechanical stimuli, consistent with elevated sensory gain (Castellanos et al., 2020). Cell-type-specific analyses further refine this role, showing that **TRESK** deletion selectively increases the intrinsic excitability of small-diameter nociceptors, particularly non-peptidergic IB4-negative neurons, while sparing IB4-positive populations (Guo et al., 2019). In rat DRG, knocking down **TRESK** increased neuronal excitability indicated by depolarized RMP, reduced rheobase current, and increased AP numbers (Yang et al., 2018). On the contrary, overexpressing **TRESK** in DRG neurons inhibited neuronal hyperexcitability (Yang et al., 2018). A similar selectivity is observed in TG neurons, where **TRESK** loss preferentially enhances excitability in IB4-negative afferents (Guo et al., 2019). Consistent with these findings, **TRESK** has also been implicated in the control of TG neuron excitability (Park et al., 2018). Beyond nociceptors, **TRESK** also exerts a critical inhibitory role in MrgprA3-positive pruriceptive neurons, linking also its function to itch regulation (Llimós-Aubach et al., 2025; Guo et al., 2019).

#### 4.2.2 Effects of increasing K2P channel expression

We have found only one study that directly examined the impact of increased expression or genetically driven GOF of K2P channels on neuronal excitability in the context of nociception. In this study, overexpression of **TRESK** in cultured mouse TG neurons increased background K^+^ currents, reduced input resistance, and elevated the threshold for action potential initiation, resulting in decreased excitability and suppression of capsaicin-evoked firing (Guo and Cao, 2014).

Although limited, this evidence supports the idea that enhancing K2P channel expression or activity increases leak potassium conductance, leading to membrane hyperpolarization and reduced neuronal excitability, ultimately producing antinociceptive effects. In this context, genetic approaches based on overexpression or GOF variants represent a valuable strategy to validate K2P channels as therapeutic targets and to assess the potential consequences of their activation beyond pain pathways, particularly given the current lack of selective pharmacological activators for several K2P subtypes.

### 4.3 Effects of K2P channel modulation on pain behaviors in mouse

**FIGURE 9:**
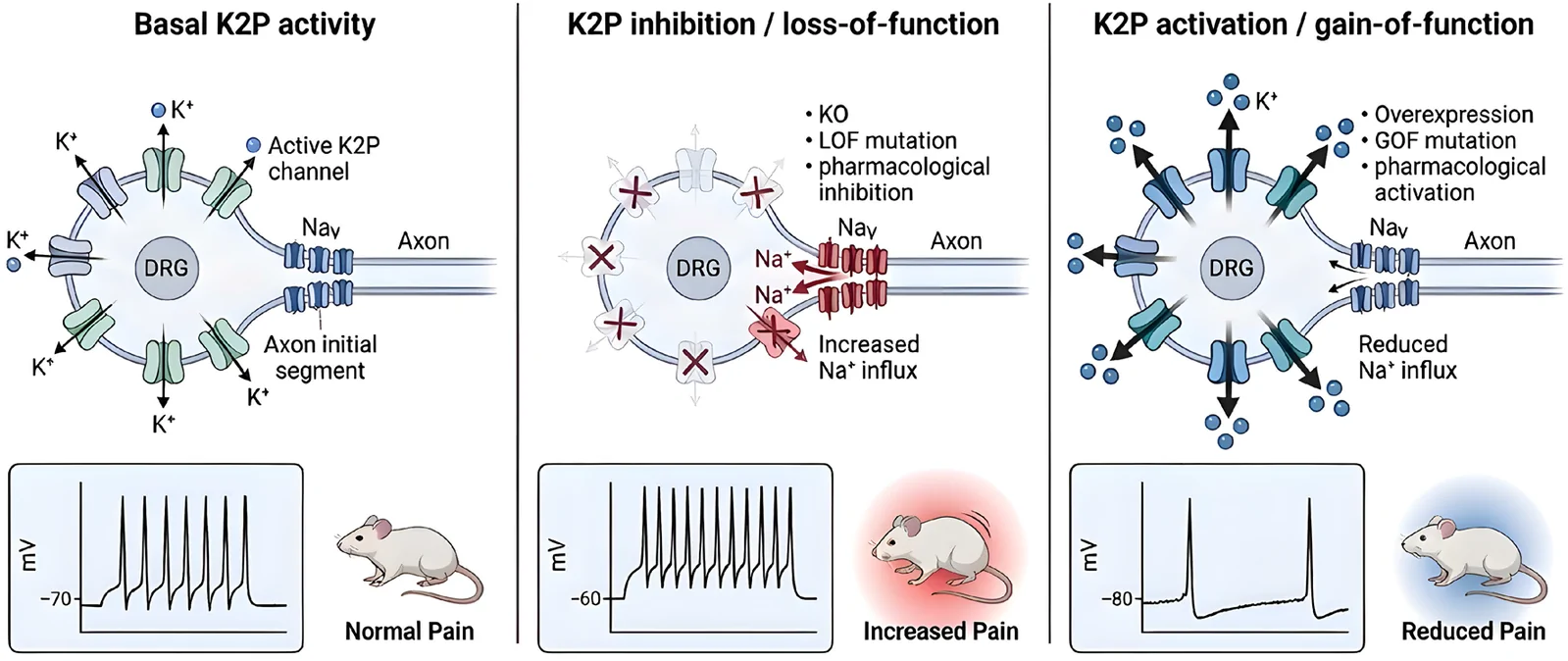
Impact of K2P channel regulation on sensory neuron excitability and pain sensitivity. K2P channels act as negative regulators of sensory neuron excitability and can therefore be considered antinociceptive channels. Under basal conditions, they help maintain the membrane potential (mV) in a hyperpolarized state and limit neuronal firing. Consequently, genetic KO, LOF mutations, or pharmacological inhibition of K2P channels increases neuronal excitability and firing frequency, leading to enhanced pain sensitivity. Conversely, increased K2P channel expression or activity, through GOF mutations or pharmacological activation, decreases neuronal excitability and firing, resulting in reduced pain sensitivity or even analgesia. Abbreviations: K2P: two-pore potassium channel, Na_V_: voltage-dependent sodium channel, KO: knockout, LOF: loss-of-function, GOF: gain-of-function, mV: millivolt.

Because K2P channels generate background potassium currents that stabilize the RMP and limit neuronal firing, modulation of their activity is expected to strongly influence pain-related behaviors *in vivo*. In sensory neurons, reduction or loss of K2P channel function generally increases membrane excitability, thereby promoting nociceptive transmission and enhancing pain sensitivity (Figure 9, left panel) (Luo et al., 2021; Li and Toyoda, 2015). Consequently, KO or LOF animal models are typically associated with increased pain phenotypes, whereas K2P overexpression or expression of GOF variants is expected to reduce nociceptor excitability and potentially produce analgesic effects (Figure 9, middle and right panels).

However, the behavioral consequences of K2P channel modulation depend on the specific channel subtype involved, as different K2P channels are expressed in distinct sensory neuron populations and are differentially regulated by mechanical, thermal, chemical, or inflammatory stimuli. As a result, the modulation of K2P channels can differentially affect mechanical, thermal, or osmotic sensitivity under acute conditions, as well as in models of acute or chronic inflammatory pain, chronic neuropathic pain, migraine-associated pain, or itch-related sensory disorders.

#### 4.3.1 Knock-out and loss-of-function mouse models

To date, most genetic studies have relied on global or constitutive KO mouse models. Although these approaches have been applied to several, but not all, K2P channels, they have revealed distinct and sometimes divergent phenotypes depending on the pain modality assessed (Table 2).

**TABLE 2:** Pain phenotypes associated with genetic deletion or loss-of-function of K2P channels. Summary of the pain phenotypes reported in genetic models lacking functional K2P channels (KO or LOF models). For each KCNK gene and corresponding K2P channel subtype, the table summarizes the main alterations in nociceptive behaviors, the sensory modalities affected, and the associated pain conditions. Overall, reduced K2P channel activity is frequently associated with increased neuronal excitability and enhanced pain sensitivity, supporting their role as endogenous negative regulators of nociceptive signaling.

Gene	Channel	KO/KD/LOF phenotype	Main modalities affected	References
*Kcnk1*	**TWIK1**	↑ Mechanical threshold ↑ persistence of neuropathic hypersensitivity after CCI	Mechanical, inflammatory, neuropathic	Yang et al. (2026)
*Kcnk2*	**TREK1**	Heat hypersensitivity Mechanical allodynia Altered osmotic nociception ↑Cold sensitivity	Thermal, mechanical, inflammatory, neuropathic migraine	Royal et al. (2019), García et al. (2021b), Alloui et al. (2006), Noël et al. (2009)
*Kcnk3*	**TASK1**	↑ Heat sensitivity	Thermal	Linden et al. (2006)
*Kcnk4*	**TRAAK**	Heat hypersensitivity Cold perception	Thermal, cold	Alloui et al. (2006), Noël et al. (2009)
*Kcnk9*	**TASK3**	↑ Cold sensitivity ↑ inflammatory and neuropathic pain ↑ Itch	Thermal, inflammatory, neuropathic, itch	Morenilla-Palao et al. (2014), Liao et al. (2019), Luo et al. (2026)
*Kcnk10*	**TREK2**	Altered warm/cool perception Oxaliplatin-induced cold allodynia ↑ Inflammatory pain	Thermal, inflammatory, osmotic, migraine	Acosta et al. (2014), Royal et al. (2019), Pereira et al. (2014)
*Kcnk12*	**THIK2**	↑ Heat and mechanical sensitivity Enhanced inflammatory pain	Thermal, mechanical, inflammatory	Gilbert et al. (2026)
*Kcnk18*	**TRESK**	↑ Cold and mechanical sensitivity Migraine LOF mutations ↑ Itch	Cold, mechanical, neuropathic, migraine, itch	Lafrenière et al. (2010), Royal et al. (2019), Yang et al. (2018), Li et al. (2019)

Abbreviations: KO: knockout, LOF: loss-of-function, DRG: dorsal root ganglion.

Thermal nociception: Studies using KO mouse models have established a central role for **TREK1**, **TRAAK** and **TREK2** in thermal nociception (Alloui et al., 2006; Noël et al., 2009). **TREK1**-deficient mice exhibit hypersensitivity to moderately noxious heat (46 °C–50 °C), with reduced withdrawal latencies, while responses at higher temperatures remain unchanged, indicating that **TREK1** selectively constrains nociceptor excitability within a defined thermal range, likely by opposing TRPV1-driven depolarization. A similar phenotype is observed in **TRAAK** KO mice, which also display heat hyperalgesia in this temperature range. In contrast, **TREK2** appears to regulate non-aversive thermal perception, contributing to the detection of warm (40 °C–46 °C) and moderately cool (20 °C–25 °C) temperatures, and is additionally implicated in oxaliplatin-induced cold allodynia (Pereira et al., 2014). Together, these channels exhibit complementary and partially overlapping functions in thermosensation, a concept further supported by double KO studies. While **TRAAK**-deficient mice show heat hypersensitivity between 46 °C and 50 °C, combined deletion of **TREK1** and **TRAAK** broadens this phenotype, extending hyperalgesia to lower temperatures (44 °C–50 °C) suggesting that **TREK** and **TRAAK** have complementary roles in thermosensation. Similarly, although **TRAAK** KO alone does not affect cold sensitivity, **TREK1/TRAAK** double KO mice display enhanced responses to cooling (20 °C–10 °C), demonstrating a cooperative role in cold perception. Consistent with this, both channels are involved in chemotherapy-induced neuropathy, as oxaliplatin reduces their expression in DRG neurons and **TREK1/TRAAK**-deficient mice fail to develop cold hypersensitivity under these conditions (Descoeur et al., 2011). Other K2P channels also contribute to thermosensory processing. Deletion of **TRESK** leads to a marked increase in cold sensitivity with only minor effects on heat perception (Weir et al., 2019; Castellanos et al., 2020; Guo and Cao, 2014; Chae et al., 2010). Although not directly temperature-gated, **TASK1** and **TASK3** also modulate thermal sensitivity: **TASK1** KO mice exhibit increased responses to noxious heat, whereas **TASK3** deletion enhances cold sensitivity, consistent with its enrichment in TRPM8-positive cold-sensitive neurons (Morenilla-Palao et al., 2014; Linden et al., 2006). Finally, more recent work has demonstrated that **THIK2** also contributes to thermal nociception, as its deletion leads to increased heat sensitivity, although its role in cold perception remains to be determined (Gilbert et al., 2026). Regardless of whether a K2P channel is inherently sensitive to temperature and directly participates in thermotransduction within the fibers where it is expressed—together with other thermosensitive proteins such as TRP channels—or instead primarily modulates neuronal excitability in thermosensitive neurons without being itself gated by temperature, K2P channels still play a critical role in the processing of thermal signals.

Mechanical nociception: Among K2P channels, only **TREK/TRAAK** exhibit intrinsic mechanosensitivity (Brohawn, 2015; Lengyel et al., 2021). Consistent with this property, **TREK1** KO mice show increased mechanical sensitivity, reflected by a significant reduction in withdrawal thresholds to von Frey stimulation, indicative of mechanical allodynia (Alloui et al., 2006). Expression patterns further suggest that **TREK2** may also contribute to the modulation of mechanical sensitivity, although its precise role remains less well defined. In parallel, deletion of **TRESK** leads not only to cold hypersensitivity but also to a marked increase in mechanical sensitivity (Weir et al., 2019; Guo et al., 2019; Castellanos et al., 2020). Interestingly, **THIK2** has also been implicated in somatosensory processing, as its deletion results in enhanced mechanical sensitivity, with reduced paw withdrawal thresholds compared to WT animals (Gilbert et al., 2026). Finally, **TWIK1** KO mice were recently shown to exhibit reduced sensitivity to innocuous and low-intensity mechanical stimuli, whereas responses to noxious mechanical stimulation are preserved (Yang et al., 2026). These results indicate that **TWIK1** contributes selectively to baseline mechanical nociception and mechanosensation under physiological conditions. Whether a K2P channel is intrinsically mechanosensitive and directly contributes to mechanotransduction within the fibers in which it is expressed—alongside other mechanosensitive proteins such as PIEZO, TMEM120A/TACAN, STOML3 (stomatin-like protein 3), and integrins—or instead acts as a regulator of neuronal excitability in mechanosensitive neurons without being itself mechanically gated, K2P channels nonetheless play a key role in the transduction of mechanical signals.

Osmotic nociception: Evidence supporting the involvement of K2P channels in osmotic nociception primarily arises from genetic studies, with **TREK1** emerging as a key regulator. **TREK1** KO mice display reduced sensitivity to strongly hypertonic stimuli (e.g., 10% NaCl), while responses to milder osmotic challenges (2% NaCl) remain comparable to WT controls. Following inflammatory sensitization with prostaglandin E2 (PGE2), these differences become more pronounced, as **TREK1** KO animals exhibit markedly diminished responses to both hyper- and hypo-osmotic stimuli and fail to develop normal osmotic hypersensitivity (Alloui et al., 2006). Consistent with a role for **TREK1**, double KO mice lacking both **TREK1** and **TRAAK** show a profound loss of osmotic sensitivity, whereas **TRAAK** single KO animals do not differ from WT controls (Noël et al., 2009). In addition, **TREK2** has been implicated in the inflammatory modulation of osmotic nociception, as **TREK2**-deficient mice fail to develop normal sensitization to osmotic stimuli following PGE2 treatment. Collectively, these findings indicate a coordinated contribution of **TREK** subfamily members to osmotic pain, particularly under sensitized conditions. However, as most K2P channels are not intrinsically osmosensitive, their involvement likely relies on the regulation of neuronal excitability rather than direct stimulus transduction, and evidence for the participation of other K2P family members remains limited.

Inflammatory pain: **TREK1** contributes to dampening persistent inflammatory pain, as demonstrated in formalin and carrageenan models (García et al., 2021b; Boyer et al., 2025). **TREK1** KO mice have increased mechanical hyperalgesia during inflammation (Noël et al., 2009). For example, **TREK1** KO mice display higher mechanical hyperalgesia than WT following intraplantar injection of carrageenan (Alloui et al., 2006). Similarly, siRNA-mediated KD of **TREK2** in rats subjected to CFA-induced inflammatory pain results in enhanced nociceptive behavior, characterized by an increased number of pain-related responses (Acosta et al., 2014). **TRESK** KO mice subjected to CFA-induced inflammation in the hind paw do not exhibit significant differences in either mechanical or thermal hypersensitivity compared with WT controls, except for a transient increase in baseline mechanical sensitivity (Castellanos et al., 2020). Overall, these findings suggest a limited contribution of **TRESK** to inflammatory pain processing. In inflammatory and neuropathic pain models, **TASK3** KO mice displayed aggravated mechanical allodynia, spontaneous pain, and thermal hyperalgesia (Liao et al., 2019). **THIK2** KO mice display both mechanical and heat hypersensitivity under inflammatory conditions induced by intraplantar injection of CFA (Gilbert et al., 2026). This nociceptive phenotype is also reflected in an increased number of pain-related behaviors following formalin (2%) administration.

Neuropathic pain: Neuropathy is a well-established cause of cold allodynia (Bennett and Xie, 1988). A decreased sensitivity to acetone is observed in the **TREK1** KO animals when mice are made mononeuropathic, suggesting that **TREK1** then becomes involved in cold sensing (Noël et al., 2009). No significant difference in cold responsiveness was observed between neuropathic **TRAAK**-deficient and WT mice. In contrast, neuropathic **TREK1/TRAAK** double KO mice exhibited pronounced cold hypersensitivity compared with both WT and **TRAAK**-deficient animals at 15 °C and 20 °C (Alloui et al., 2006). Because a robust downregulation of **TRESK** was reported in different models of neuropathic pain, including complete axotomy, spared nerve injury, and spinal nerve ligation sensitivity of **TRESK** KO animals were compared to WT in different neuropathic models (Tulleuda et al., 2011; Hwang et al., 2015; Zhou et al., 2013; Zhou et al., 2017). However, no significant differences were observed between WT and **TRESK** KO mice in the development of mechanical or thermal hypersensitivity in a nerve-cuffing model of neuropathic pain. Interestingly, **TRESK**-deficient mice exhibited cold allodynia comparable to that induced by oxaliplatin in WT animals, with no further increase in cold hypersensitivity following chemotherapy treatment. Whether **TRESK** deletion induces compensatory cellular or molecular adaptations affecting cold-sensitive nociceptor excitability remains to be determined. Unexpectedly, a recent study showed that, following CCI, **TWIK1** KO mice develop an initial mechanical hypersensitivity comparable to that of WT mice but exhibit a markedly accelerated recovery, thereby limiting the persistence of neuropathic hypersensitivity (Yang et al., 2026). This unexpected phenotype suggests that, unlike other K2P channels, **TWIK1** may promote the maintenance of neuropathic pain through mechanisms extending beyond its canonical function, potentially involving injury-induced transcriptional remodeling of sensory neurons. Further work will be required to confirm this unexpected pronociceptive role of **TWIK1** and clarify the underlying mechanisms.

Migraine-like phenotypes: Although no study has directly linked reduced **TRESK** expression *per se* to migraine, multiple LOF mutations in *KCNK18* have been associated with familial migraine with aura. The first identified mutation, F139WfsX24, produces a truncated, non-functional channel that inhibits WT **TRESK** current in a dose-dependent manner, thereby exerting a dominant-negative effect (Lafrenière et al., 2010). Other variants, such as W101R and Y121LfsX44, similarly impair channel activity and may contribute to migraine susceptibility (Royal et al., 2019; Imbrici et al., 2020). Importantly, the pathogenicity of F139WfsX24 extends beyond a simple LOF. This frameshift mutation gives rise, through alternative translation, to two protein fragments, termed MT1 and MT2, that remain functionally active. The MT1 fragment, corresponding to the short N-terminal region of **TRESK**, exerts dominant-negative effect on WT **TRESK** whereas MT2, a novel protein generated by the frameshift, inhibits not only **TRESK** but also the related K2P channels **TREK1** and **TREK2** through heteromeric interactions (Royal et al., 2019). In an animal model of migraine, the injection with the NO-donor isosorbide dinitrate (ISDN), a known migraine trigger, resulted in the increased sensitivity to mechanical stimuli (mechanical allodynia, a common symptom of migraine headache) in animals deficient in both **TREK1** and **TREK2**, compared to their WT littermates (Royal et al., 2019). This broader suppression of background K^+^ conductance increases TG neuron excitability and induces migraine-like mechanical hypersensitivity *in vivo*, whereas the MT1 truncated protein alone is insufficient to reproduce this phenotype. These findings help reconcile earlier inconsistencies, as not all *KCNK18* LOF variants are associated with migraine, highlighting the specific contribution of dominant-negative and cross-channel effects (Guo and Cao, 2014; Andres-Enguix et al., 2012). Consistently, pharmacological modulation of **TRESK**-related pathways alleviates hypersensitivity in rodent migraine models, supporting K2P channel networks as promising therapeutic targets for migraine and trigeminal pain (Verkest et al., 2021).

Itchy phenotypes: Deletion of the **TASK3** channel has been shown to enhance itch sensitivity, supporting its role as an important negative regulator of pruriceptor excitability (Luo et al., 2026). Sensory neuron-specific deletion of **TASK3** in DRG neurons increased scratching behaviors in response to pruritogens and enhanced the activity of GRP-positive neurons in the spinal dorsal horn. Along the same line, genetic ablation of **TRESK** also increased itch-related behaviors (Llimós-Aubach et al., 2025). **TRESK**-deficient mice displayed enhanced firing of MrgprA3-expressing pruriceptors and increased scratching responses following chloroquine injection, whereas histamine-dependent itch responses were largely unaffected. In addition, **TRESK** deletion exacerbated chronic itch phenotypes in several mouse models, including allergic contact dermatitis, dry skin, and imiquimod-induced psoriasiform dermatitis, leading to earlier onset and more severe scratching behaviors. Together, these recent studies indicate that **TASK3** and **TRESK** act as endogenous brakes limiting itch signaling pathways and pruriceptor hyperexcitability.

#### 4.3.2 Overexpression or gain-of-function mouse models

Very few studies have explored strategies based on overexpressing K2P channels in sensory neurons or expressing GOF versions of these channels. Yet, such approaches provide a powerful way to evaluate whether selective activation of a given K2P channel subtype can reduce pain-related behaviors.

Several examples supporting this concept can nevertheless be found in the literature. For instance, viral restoration of **TWIK1** expression in DRG neurons of SNL-induced neuropathic pain animals markedly reduced both mechanical and thermal hypersensitivity without affecting acute nociception (Mao et al., 2017). Similarly, overexpression of **TRESK** channels reduces the excitability of TG neurons (Guo and Cao, 2014). In another model, overexpression of the **TRESK** channel in cancer-associated pain models was also shown to reduce neuronal hyperexcitability and attenuate hyperalgesia (Liu et al., 2021; Yang et al., 2018). In rodent CCI models of the infraorbital nerve, **TRESK** overexpression reduced mechanical allodynia and cognitive impairment, whereas **TRESK** KD induced pain behaviors (Li et al., 2019). Consistently, **TRESK** overexpression in DRG neurons attenuated tumor-induced neuronal hyperexcitability and pain hypersensitivity in a bone metastasis model (Yang et al., 2018).

In a related approach, *in vivo* delivery of constitutively active **TREK1** mutants (S300A/S333A) by intrathecal lipofection effectively reversed tactile allodynia in neuropathic rats, further supporting the concept that increasing K2P channel activity dampens nociceptor excitability (García et al., 2021b).

### 4.4 Peripheral versus central contributions of K2P channels to pain

Distinguishing the respective contributions of peripheral and central K2P channels to pain processing is essential for understanding their physiological roles and for developing targeted analgesic therapies. However, this remains a major challenge because most experimental approaches do not allow these two components to be clearly dissociated. Several K2P channels are expressed not only in primary sensory neurons but also throughout the spinal cord and in multiple supraspinal structures involved in nociceptive processing, including regions associated with sensory-discriminative, affective, and descending modulatory pathways (Medhurst et al., 2001; Talley et al., 2001). While this expression pattern supports a potential contribution of K2P channels to central pain processing, most functional evidence has been obtained in DRG and TG neurons, where their role in regulating sensory neuron excitability is firmly established (Weir et al., 2019; Guo et al., 2019; Royal et al., 2019; Andres-Bilbe et al., 2020; Yang et al., 2018; Alloui et al., 2006; Noël et al., 2009; Pereira et al., 2014; Pettingill et al., 2019; Castellanos et al., 2020; Park et al., 2018; Guo and Cao, 2014).

Evidence supporting a direct role of central K2P channels remains limited. Intrathecal administration of **TREK1** modulators reduces nociceptive behaviors in formalin-induced pain models, suggesting a spinal site of action (Alloui et al., 2006). However, intrathecal delivery cannot distinguish between effects on dorsal horn neurons, spinal glial cells, or the central terminals of primary afferent neurons, all of which may express **TREK1**. Likewise, although **THIK1** regulates microglial ramification and inflammatory responses, no direct evidence currently demonstrates that microglial K2P channels contribute to pain processing (Madry et al., 2018; Izquierdo et al., 2021). A supraspinal analgesic role for **TREK1** was also proposed following pharmacological activation in the anterior cingulate cortex; however, this study has since been retracted and therefore cannot be considered reliable evidence supporting a central analgesic function (Peng et al., 2022).

Interpretation of genetic studies is similarly complicated. Most nociceptive phenotypes described for **TREK1**, **TREK2**, **TRAAK**, **TRESK**, **TASK1, TASK3** and **THIK2** have been obtained using constitutive global KO (Acosta et al., 2014; Lafrenière et al., 2010; Gilbert et al., 2026; Morenilla-Palao et al., 2014; Liao et al., 2019; Royal et al., 2019; García et al., 2021a; Yang et al., 2018; Alloui et al., 2006; Noël et al., 2009; Pereira et al., 2014; Luo et al., 2026; Linden et al., 2006; Li et al., 2019). Because these models simultaneously affect peripheral sensory neurons, their central terminals, spinal neurons, glial cells and supraspinal circuits, the resulting behavioral phenotypes cannot be unequivocally attributed to either peripheral or central mechanisms.

Very recently, selective viral-mediated inhibition of **TWIK1** in the spinal cord was shown to alter mechanical nociception in a neuropathic pain model (Yang et al., 2026). These findings suggest that **TWIK1** expressed in GABAergic inhibitory interneurons of the spinal cord plays a predominant role in regulating these sensory functions. Importantly, this mechanism contrasts with the antinociceptive role generally attributed to peripheral K2P channels. In spinal inhibitory interneurons, **TWIK1** appears to act as a brake on inhibitory neurotransmission, thereby contributing to persistent mechanical hypersensitivity in neuropathic pain. These observations underscore the importance of distinguishing not only between the peripheral and central functions of K2P channels, but also between their roles in distinct neuronal populations, including inhibitory and excitatory spinal neurons. Such cellular and anatomical specificity will be critical for the development of K2P-targeted analgesic therapies, as indiscriminate modulation of channel activity may produce markedly different—or even opposing—effects in the PNS and CNS.

Overall, although anatomical and physiological evidence supports a potential contribution of central K2P channels to pain modulation, their specific roles within spinal and supraspinal circuits remain unresolved. The limited cellular selectivity of currently available pharmacological modulators and the inability of conventional behavioral assays to dissociate peripheral transduction, spinal transmission and supraspinal pain perception further complicate the interpretation of existing studies. Definitive evidence will require cell type- and region-specific genetic manipulations, combined with electrophysiological and behavioral approaches capable of independently interrogating peripheral, spinal and supraspinal mechanisms.

## 5 Pharmacological modulation of pain by K2P channels

### 5.1 Modulators of TREK1/TREK2/TRAAK

A growing number of small-molecule activators of **TREK** and **TRAAK** channels have been identified over the past two decades, progressively strengthening the link between pharmacological activation of these K2P channels and analgesia (Ávalos Prado et al., 2022; Decher et al., 2021; Kamuene et al., 2021; Kim, 2005; Lesage, 2003; Mathie and Veale, 2015; Natale et al., 2021).

#### 5.1.1 TREK/TRAAK activators

Riluzole, a neuroprotective compound and one of the earliest known **TREK1/TRAAK** activators, produces anti-nociceptive and anti-allodynic effects in neuropathic pain models and enhances inhibitory synaptic transmission in the dorsal horn (Duprat et al., 2000; Taiji et al., 2021). High-throughput screening approaches, particularly thallium-flux–based assays, have led to the identification of additional **TREK1** activators, including RNE28 and LPS2336 (Boyer et al., 2025; Busserolles et al., 2020). Systemic administration of these compounds produces robust analgesic effects in mice, although accompanied by notable side effects, thereby nonetheless underscoring the therapeutic potential of peripherally targeting **TREK** channels for pain management (Boyer et al., 2025; Busserolles et al., 2020). Using similar screening approaches, ONO-2920632 (VU6011887) was identified as a selective **TREK2** activator that displays strong blood–brain-barrier permeability and alleviates nocifensive behaviors in the acetic-acid writhing test in rats and mice (Yashiro et al., 2025). Additional evidence for **TREK2**-mediated analgesia comes from orofacial sensory neurons, where baclofen induces membrane hyperpolarization and increases leak K^+^ currents in IB4-positive trigeminal neurons, suggesting a functional interplay between GABA_B_ receptors and **TREK2** channels in modulating pain sensitivity (Chang et al., 2021). In migraine models in rodents, activation of **TREK1** and **TREK2** by the compound ML67-33 reduces trigeminal neuronal hyperexcitability and suppresses headache-like behaviors triggered by NO (Ávalos Prado et al., 2021; Bagriantsev et al., 2013). Other TREK activators have shown promising cellular effects, including GI-530159—a **TREK1/2** opener that hyperpolarizes cultured mouse DRG neurons expressing these channels (Lou et al., 2018). C3001a targets **TREK1** and **TREK2** channels and was shown to alleviate spontaneous pain and cold hyperalgesia in mouse. The same compound was tested on a mouse model of acute pancreatitis, and also found to reduce mechanical allodynia and inflammation (Qiu et al., 2020). Endogenous lipid metabolites can also modulate **TREK** activity: arachidonic-acid derivatives such as 11-deoxy-prostaglandin F2α enhance **TREK2** currents in heterologous systems and increase K^+^ currents in IB4-positive DRG neurons, suggesting physiological relevance in nociceptive signaling (Dadi et al., 2017). More recently, ML335, a **TREK1/2** activator, has been incorporated into a chemogenetic strategy enabling covalent locking of modified **TREK** channels in an open state, offering precise inhibition of neuronal activity in defined hippocampal circuits and providing an innovative tool to dissect the contribution of TREK-expressing fibers to pain pathways (Deal et al., 2024). In a recent study, the same group demonstrated that the K2P channel **TRAAK** can be directly activated by UV-A illumination, revealing an optical strategy to modulate nociceptor excitability and pain signaling (Bied et al., 2026). Using 365 nm light exposure, the authors showed that activation of endogenous **TRAAK** channels in peripheral nociceptor endings produced a robust and long-lasting analgesic effect in both acute and chronic pain models, including neuropathic pain conditions. Mechanistically, UV exposure induced oxidation of a methionine residue within the **TRAAK** channel, stabilizing its active conformation and thereby enhancing potassium leak currents and reducing nociceptor excitability. Although this non-invasive and drug-free approach is unlikely to be directly translatable to humans because human **TRAAK** channels are not UV-sensitive, it nevertheless represents an innovative proof-of-concept highlighting the therapeutic potential of K2P channel activation for pain management in veterinary medicine and animal experimentation. Pharmacological activation of **TREK1**, **TREK2** and **TRAAK** channels with riluzole or BL-1249 relaxes colonic smooth muscle, highlighting their role in gastrointestinal function (Ma et al., 2018). In addition, **TREK1** is expressed in colorectal visceral afferents, and its expression and stretch-activated currents are reduced during experimental colitis, suggesting a role in colorectal mechanosensitivity (La et al., 2011). Although these findings implicate mechanosensitive K2P channels in colorectal sensory physiology, there is currently no direct evidence linking K2P channels to visceral pain.

#### 5.1.2 TREK/TRAAK inhibitors

Although **TREK** and **TRAAK** channel inhibitors are not suitable for clinical application, they have provided powerful experimental tools to causally link the activity of these K2P channels to pain processing. ML365, a non-selective inhibitor of K2P channels, has enabled the development of an innovative photosensitive pronociceptive tool, LAKI-ML365 (light-activated K^+^ inhibitor), which permits reversible inhibition of K2P channel activity. Using this opto-pharmacological strategy, researchers demonstrated *in vivo* that acute inhibition of **TREK1, TREK2** and **TRESK** channels directly enhances nociceptive behaviors, providing compelling evidence for their physiological role as endogenous “brakes” on pain signaling (Landra-Willm et al., 2023). Additional insights arise from neuropathic pain models, such as the prediabetic high-energy diet–induced DPNP (diabetic peripheral neuropathic pain) rat model, where mechanical allodynia and thermal hyperalgesia are associated with sensory neuron hyperexcitability, upregulation of Sortilin, and concomitant downregulation of **TREK1** and **TREK2** expression in DRG neurons. This pathogenic mechanism is further supported by the observation that local injection of Spadin—a Sortilin-derived peptide known to inhibit **TREK** channels—induces acute hyperalgesia when administered into the DRG of affected rats. Conversely, intra-ganglionic delivery of a **TREK1/2** activator reverses both mechanical and thermal hypersensitivity, reinforcing the notion that diminished **TREK** channel function directly contributes to neuropathic pain states (Mazella et al., 2010; Sun et al., 2024).

Together, these pharmacological studies highlight the strong non-opioid analgesic potential of **TREK** and **TRAAK** channel activation. However, the current generation of compounds lacks sufficient isoform specificity, making it difficult to disentangle the respective contributions of **TREK1**, **TREK2**, and **TRAAK** and increasing the likelihood of off-target effects. This limitation is particularly relevant given the broader peripheral and central distribution of **TREK** channels compared with the more neuronally restricted expression of **TRAAK**, suggesting that isoform-selective targeting—potentially favoring **TRAAK**—may represent a safer therapeutic approach with fewer systemic side effects.

### 5.2 Modulators of TASK1/TASK3

TASK1 and TASK3 activators: To date, two small-molecule activators of **TASK3** channels have been reported for their analgesic potential *in vivo*. Terbinafine, a relatively selective **TASK3** activator, was the first compound used to explore the contribution of **TASK3** to pain processing (Wright et al., 2017). Although its use is limited by significant peripheral and central side effects, including gastrointestinal disturbances, hepatotoxicity, and dizziness, studies using terbinafine suggested a role for spinal **TASK3** channels in the modulation of inflammatory and neuropathic pain. They also show that intrathecal pretreatment with the activator terbinafine could reduce formalin-induced flinching and nociceptive hyperalgesia in rats with neuropathy (García et al., 2019). More recently, the identification of CHET3 provided a more compelling pharmacological tool to assess the analgesic relevance of **TASK3** activation (Liao et al., 2019). Systemic administration of CHET3 produces robust antinociceptive effects in multiple mouse models of acute, inflammatory, and neuropathic pain, reducing thermal hyperalgesia, mechanical allodynia, and particularly cold hypersensitivity, with greater efficacy than pregabalin in a neuropathic pain model. The analgesic action of CHET3 is strictly **TASK3** dependent, as evidenced by its complete loss of efficacy in **TASK3**-deficient mice. Pharmacokinetic studies further indicate that CHET3 acts predominantly at peripheral sites, consistent with a mechanism involving reduced excitability of peripheral sensory neurons rather than CNS effects. Notably, despite CHET3 eliciting responses in fewer than 20% of DRG neurons, selective activation of **TASK3**-containing channels results in a remarkably strong analgesic outcome, underscoring the therapeutic relevance of this target. Pharmacological studies further support the involvement of **TASK3** in itch modulation. Activation of **TASK3** markedly reduced both acute and chronic itch behaviors in mice, highlighting its potential as a therapeutic target for pruritus (Luo et al., 2026). These results suggest that pharmacological enhancement of **TASK3** activity may represent an effective strategy to suppress pruriceptor hyperexcitability and alleviate chronic itch.

TASK1 and TASK3 inhibitors: Inhibition of **TASK1** and **TASK3** channels using halothane and alkalinization induced a slight membrane depolarization and a significant increase in the firing frequency of nodose ganglion neurons (Park et al., 2018). These findings indicate these channels contribute to maintaining basal electrical tone and act to limit neuronal hyperexcitability within the vagal system. Intrathecal administration of ML365 and PK-THPP—two blockers for **TASK1** and **TASK3**—exacerbates both hyperalgesia and allodynia induced by formalin injection in a murine model of inflammatory pain (Coburn et al., 2012; Flaherty et al., 2014). These inhibitors also abolish the analgesic effects of terbinafine in a neuropathic pain model, further supporting a pronociceptive consequence of **TASK1** and/or **TASK3** channel blockade (García et al., 2019). Notably, central responses may vary, given the expression of these channels in respiratory and arousal circuits. In contrast, pruritogens such as histamine and chloroquine were found to directly inhibit **TASK3**-mediated potassium currents, thereby increasing the excitability of a subset of sensory neurons specialized in itch transmission (Luo et al., 2026).

### 5.3 Modulators of TRESK

There are few pharmacological tools modulating **TRESK** channel. Isobutylalkyl amide, an inhibitor of **TRESK**, could evoke flinching and licking behaviors in rats (Tulleuda et al., 2011). Among the currently available compounds, **cloxyquin** has emerged as one of the best characterized **TRESK** activators. Pharmacological enhancement of **TRESK** activity with cloxyquin significantly reduced both acute and chronic itch behaviors in WT mice, whereas these effects were absent in **TRESK** KO animals, supporting a specific **TRESK**-dependent mechanism (Llimós-Aubach et al., 2025). In these studies, **TRESK** activation decreased the excitability of pruriceptive neurons involved in histamine-independent itch pathways, further supporting the role of **TRESK** as an endogenous brake limiting sensory neuron firing.

### 5.4 Analgesics, anesthetics, anti-inflammatory and anti-oxidant molecules

Several widely used drugs modulate K2P channels and have been studied in pain models, often as off-target effects.

Morphine: Systemic administration of morphine in both WT and **TREK1** KO mice, using neuropathic and postoperative pain models, has shown that the presence of the **TREK1** channel enhances the analgesic effect of this opioid (Devilliers et al., 2013). Moreover, **TREK1** is not involved in the typical side effects induced by morphine, such as dependence, respiratory depression, or constipation. These findings strengthen the rationale for developing specific activators of this channel to improve treatment efficacy while limiting adverse effects.

Volatile and gaseous anesthetics: Certain K2P channels are strongly modulated by volatile anesthetics, including isoflurane, sevoflurane, halothane, diethyl ether and chloroform, as well as by gaseous anesthetics such as nitrous oxide (N_2_O), xenon and cyclopropane (Natale et al., 2021; Mathie et al., 2021). Interestingly, volatile anesthetics exhibit marked subtype-dependent effects among K2P channels, with differences in both sensitivity and direction of modulation. Among K2P channels, **TREK1** and **TREK2**, but not **TRAAK**, are robustly activated by several clinically used volatile anesthetics. **TREK1** displays particularly high sensitivity to isoflurane, halothane, diethyl ether and chloroform (Patel et al., 1999). Mechanistically, a hydrophobic pocket located at the interface of the transmembrane domains of **TREK1** has been proposed as a potential binding site for volatile anesthetics, while the intracellular C-terminal domain contributes to anesthetic-dependent activation through mechanisms involving phosphorylation-dependent regulation (Patel et al., 1999; Gruss et al., 2004; Wague et al., 2020). **TREK1** KO mice display a reduced sensitivity to several volatile anesthetics supporting an important contribution of **TREK1** activation to anesthetic-induced neuronal silencing (Heurteaux et al., 2004). Other K2P channels also participate in anesthetic sensitivity but display more heterogeneous responses. **TRESK** is activated by most volatile anesthetics, with reported effective concentrations in the micromolar range, whereas **THIK1**, **THIK2**, **TALK1**, **TALK2, TASK2** and **TWIK2** are inhibited by these compounds (Chatelain et al., 2013; Girard et al., 2001; Kindler et al., 2003; Liu et al., 2004; Patel et al., 2000; Rajan et al., 2001; Renigunta et al., 2014). Notably, the inhibitory effect of halothane on **THIK1** was originally reflected in its nomenclature, with THIK standing for “Tandem of pore domains in a weak inward rectifying K^+^ channel inhibited by halothane” (Girard et al., 2001; Rajan et al., 2001). The TASK subfamily exhibits a particularly complex regulation by volatile anesthetics. **TASK1** and **TASK3** are both activated by halothane, whereas their response to isoflurane appears to be concentration- and channel-composition-dependent (Siroi et al., 2000). At higher concentrations, isoflurane activates **TASK3** homomeric channels and **TASK1/TASK3** heterodimers, while inhibiting **TASK1** homomeric channels, indicating that volatile anesthetics can exert bidirectional effects on TASK channel activity depending on dose and subunit assembly (Berg et al., 2004). Consistent with this functional contribution, the hyperpolarizing effects of halothane and isoflurane are markedly reduced in **TASK1/TASK3** KO models and are associated with altered anesthetic endpoints, including sedation, hypnosis and immobilization (Woo et al., 2012). Gaseous anesthetics also modulate specific K2P channels. **TREK1** and **TRESK** are activated by nitrous oxide, whereas **TASK3** appears insensitive to this anesthetic (Gruss et al., 2004; Liu et al., 2004). In addition, **TREK1** is activated by xenon and cyclopropane, further supporting its role as a major molecular target for structurally diverse anesthetic agents (Gruss et al., 2004). The modulation of K2P channels expressed in peripheral sensory neurons and pain pathways may represent an important mechanism by which anesthetics regulate nociceptive processing, with activation of **TREK/TRAAK/TRESK** channels promoting membrane hyperpolarization and reduced neuronal excitability, whereas inhibition of other subtypes such as **THIK** or **TASK2** may contribute to more complex cell-specific effects on neuronal and non-neuronal excitability.

Local anesthetics: Several clinically used local anesthetics, including lidocaine, bupivacaine, and ropivacaine, have been reported to modulate K2P channel activity, suggesting that part of their pharmacological actions may extend beyond the classical inhibition of voltage-gated sodium channels. Among K2P channels, **TREK1** is inhibited by lidocaine, bupivacaine and ropivacaine at clinically relevant concentrations (Kindler et al., 1999). The sensitivity to local anesthetics differs among members of the TREK subfamily, with **TREK1** and **TREK2** being inhibited, whereas **TRAAK** displays a much lower sensitivity, highlighting distinct pharmacological properties despite their structural similarity (Decher et al., 2021; Nayak et al., 2009; Shin et al., 2014). Beyond TREK channels, several other K2P family members, including **TASK1**, **TASK2**, **TASK3** and **TRESK** have been reported to be inhibited by amide-type local anesthetics such as bupivacaine and ropivacaine (Kindler et al., 2003; Kindler et al., 1999; Kim et al., 2000). Interestingly, this inhibition displays marked subtype specificity among TASK channels with **TASK1** showing higher sensitivity than **TASK3** channels, whereas heteromeric **TASK1/TASK3** channels display intermediate sensitivity, highlighting the importance of channel composition in determining pharmacology (Du et al., 2011). Supporting this, KO mice with global deletion of **TASK1** and **TASK3** display reduced susceptibility to local anesthetic-induced seizures, suggesting that TASK channels represent molecular targets contributing to the central neurotoxic effects of these compounds (Du et al., 2011). Since analgesic effects of local anesthetics are predominantly mediated by voltage-gated sodium channel blockade, K2P channel inhibition is likely to act as a modulatory mechanism rather than as the primary determinant of local anesthesia. Nevertheless, given the broad expression of K2P channels in sensory pathways and their key role in controlling neuronal excitability, future studies assessing local anesthetic sensitivity in additional K2P-deficient models and defining the molecular determinants governing local anesthetic–K2P channel interactions may reveal an underestimated contribution of these leak potassium channels to analgesia.

Anti-inflammatory and anti-oxidant drugs: In addition to anesthetics, several natural compounds and clinically used anti-inflammatory drugs have been shown to modulate K2P channel activity, suggesting that these channels may contribute to the pharmacological effects of diverse pain-relieving agents. Sanshool, a compound found in preparations made from Szechuan pepper and traditionally used for its anesthetic and analgesic properties, is reported to inhibit the activity of **TASK1**, **TASK3**, and **TRESK** channels expressed in small- and large-diameter TG neurons and cerebellar granular neurons (CGN) (Bautista et al., 2008). Two different studies based on the effect of herbal remedies from traditional Chinese or Native American medicines such as Kampo and aristolochic acid, reported more ambivalent outcomes, depending on the plant source of the preparations and the specific channel tested (voltage-dependent activation of **TREK** channels and inhibition of **TASK** and **TRESK** channels) (Herbrechter et al., 2020; Veale and Mathie, 2016). Analgesic and anti-inflammatory drugs such as amitriptyline, acetaminophen, ibuprofen, and nabumetone inhibit **TRESK** and/or **TREK2** channels when these are expressed and recorded in HEK cells (Park et al., 2016). The inhibitory effect of the anticonvulsant lamotrigine—and one of its derivatives—on **TREK** and **TRESK** channel activity has been proposed as a potential mechanism contributing to the treatment of pain and depression, even though a previous study had concluded that lamotrigine did not play a significant role in such therapies (Walsh et al., 2021). Although the regulation of K2P channels by antioxidant compounds remains largely unexplored, emerging evidence suggests that natural antioxidants may influence pain processing through subtype- and circuit-specific modulation of K2P channel activity. In particular, inhibition of **TASK1** channels within supraspinal pain-regulatory circuits has been proposed as a potential mechanism contributing to the analgesic effects of these compounds. In a mouse model of SNI-induced neuropathic pain, oligomeric proanthocyanidins (OPC), a class of natural antioxidant flavonoid, were shown to modulate the activity of the ventrolateral orbitofrontal cortex through a mechanism proposed to involve direct inhibition of **TASK1** channels (Gu et al., 2025). Consistent with this hypothesis, molecular docking analysis suggests that these OPC interact with the extracellular cap region of domain of **TASK1** thereby impairing channel activity.

### 5.5 Current limitations of K2P channel modulators

Despite the growing number of K2P channel activators displaying analgesic efficacy in preclinical models (Table 3), several pharmacological limitations currently hamper their therapeutic translation. Most available compounds exhibit incomplete subtype selectivity, making it difficult to distinguish the respective contributions of **TREK1**, **TREK2**, and **TRAAK** channels while increasing the likelihood of off-target effects. For example, riluzole, one of the earliest **TREK1** and **TRAAK** activators, is well known to interact with multiple ion channels and molecular targets, complicating the interpretation of its analgesic effects. Similarly, RNE28 and LPS2336 produce robust antinociceptive effects but are associated with notable side effects *in vivo*, whereas terbinafine, although relatively selective for **TASK3**, has limited translational potential because of gastrointestinal disturbances, hepatotoxicity, and central adverse effects such as dizziness. By contrast, ONO-2920632 (VU6011887) displays a more favorable selectivity profile for **TREK2** together with good blood–brain barrier permeability, while CHET3 appears particularly promising owing to its **TASK3**-dependent analgesic efficacy, predominant peripheral action, and superior performance compared with pregabalin in experimental neuropathic pain. Nevertheless, for most K2P modulators, comprehensive pharmacokinetic, safety, and long-term tolerability data remain scarce, underscoring the need for the development of highly selective compounds with improved drug-like properties before clinical translation can be considered.

**TABLE 3:** Pharmacological modulation of K2P channels *in vivo* and its impact on pain and itch behaviors. Summary of *in vivo* studies investigating pharmacological modulation of K2P channels and its consequences on pain- and itch-related behaviors. Activation of TREK, TRAAK, TASK3 and TRESK channels generally produces analgesic or antipruritic effects, whereas channel inhibition tends to increase nociceptive sensitivity. Models include acute, inflammatory, neuropathic and pruritic conditions in rodents.

Compound	K2P target	Modulation	Animal	Pain model	Effect on pain	References
RNE28	**TREK1**	Activation	Mouse	Acute, inflammatory, neuropathic	↓ Thermal and mechanical hypersensitivity	Busserolles et al. (2020)
LPS2336	**TREK1**	Activation	Mouse	Acute	↓ Nociceptive behaviors	Boyer et al. (2025)
ML67-33	**TREK1/2**	Activation	Rat/Mouse	Migraine	↓ Headache-like behaviors	Ávalos Prado et al. (2021)
C3001a	**TREK1/2**	Activation	Mouse	Neuropathic; pancreatitis	↓ Spontaneous pain ↓ Cold hyperalgesia ↓ Mechanical allodynia	Qiu et al. (2020)
Spadin	**TREK1/2**	Inhibition	Rat	Pre-diabetic neuropathy	↑ Hyperalgesia	Sun et al. (2024)
ONO-2920632	**TREK2**	Activation	Mouse	Writhing assay	↓ Nocifensive behaviors	Yashiro et al. (2025)
Riluzole	**TREK1/TRAAK**	Activation	Rat	Neuropathic	↓ Neuropathic pain ↓ Allodynia	Taiji et al. (2021)
UV-A	**TRAAK**	Activation	Mouse	Acute, inflammatory, neuropathic	Analgesic effect	Bied et al. (2026)
LAKI-ML365	**TREK/TRESK**	Inhibition	Mouse	Acute	↑ Pain behaviors	Landra-Willm et al. (2023)
OPC	**TASK1**	Inhibition	Mouse	SNI	↓ Mechanical and thermal hyperalgesia	Gu et al. (2025)
ML365	**TASK1**	Inhibition	Rat	Formalin; SNL	↑ Allodynia	García et al. (2019)
Terbinafine	**TASK3**	Activation	Rat	Formalin SNL	↓ Pain	García et al. (2019)
CHET3	**TASK3**	Activation	Mouse	Acute, chronic, itch	↓ Pain and itch	Liao et al. (2019), Luo et al. (2026)
PK-THPP	**TASK3**	Inhibition	Rat	Formalin; SNL	↑ Hyperalgesia	García et al. (2019)
Cloxyquin	**TRESK**	Activation	Mouse	Itch	↓ Itch	Llimós-Aubach et al. (2025)
IBA	**TRESK**	Inhibition	Rat	Acute	↑ Flinching and licking	Tulleuda et al. (2011)

Abbreviations: WT: wild type, KO: knockout, SNL: spinal nerve ligation, CFA: Complete Freud’s adjuvant, SNI: spared nerve injury, Ref: References.

## 6 Conclusions and perspectives

In conclusion, K2P channels have evolved from being considered passive background potassium channels to key regulators of neuronal excitability and pain signaling. By integrating mechanical, thermal, chemical, inflammatory and metabolic signals, these channels act as molecular sensors that continuously adjust nociceptor responsiveness. Genetic and pharmacological studies across inflammatory, neuropathic, cancer-related, migraine-associated and itch models have established a common principle: reducing K2P activity favors neuronal hyperexcitability and pain hypersensitivity, whereas enhancing K2P-dependent leak conductance reinforces intrinsic inhibitory mechanisms and limits excessive sensory transmission.

However, K2P channels should not be viewed as isolated regulators of nociception. Pain emerges from the integrated activity of complex molecular networks in which excitatory pathways, including Na_V_, Ca_V_, TRP, ASIC, P2XR and mechanosensitive channels, are counterbalanced by inhibitory conductance mediated by K2P and other potassium or chloride channels. In addition, membrane signaling proteins such as neurotrophic receptors (TrkA, tropomyosin receptor kinase A and RET, rearranged during transfection receptor tyrosine kinase), inflammatory receptors, chemokine receptors, CGRP and opioid receptors, as well as emerging regulators such as TACAN, continuously reshape nociceptor excitability by controlling channel activity, localization and expression (Waxman and Zamponi, 2014; Basbaum et al., 2009; Beaulieu-Laroche et al., 2020; Ji et al., 2018). Pain intensity therefore reflects the balance between multiple pro- and anti-nociceptive mechanisms rather than the function of a single molecular switch.

This complexity explains why, despite the identification of numerous promising analgesic targets and the clinical success of some ion channel- or receptor-based therapies, the search for a universal pain target has remained unsuccessful. The K2P family itself illustrates this paradigm shift. Among the 15 mammalian K2P subunits, **TREK1** and **TRESK** initially emerged as particularly attractive candidates because of their strong expression in sensory neurons and their functional links with pain and migraine (Lafrenière et al., 2010; Tulleuda et al., 2011; Alloui et al., 2006). Yet, accumulating evidence indicates that there is no unique K2P “pain channel”. Instead, transcriptomic and functional studies reveal that most K2P subtypes are expressed, to varying degrees, across peripheral and central pain pathways in rodents and humans, suggesting that different members of this family may provide therapeutic advantages depending on the pain mechanism, neuronal population targeted, and expected benefit/risk profile (Bhuiyan et al., 2024; Zeisel et al., 2018; Flegel et al., 2015; Nguyen et al., 2021; Tavares-Ferreira et al., 2022).

The next challenge is therefore not to identify the best K2P channel, but to determine which K2P channel — or combination of channels — should be targeted for a given pathological condition. Peripheral K2P modulation may be particularly suited to reduce nociceptor hyperexcitability while limiting central adverse effects, whereas spinal and supraspinal K2P channels may offer opportunities to control central sensitization and altered pain modulation. This functional diversity transforms the K2P family from a collection of individual targets into a versatile toolbox for mechanism-based analgesia.

Recent advances in single-cell transcriptomics, structural biology, genetic models and drug discovery now provide an unprecedented opportunity to define this therapeutic potential. Future pain treatments will likely move beyond the concept of a single “magic bullet” toward precision and rational polypharmacology, combining strategies that inhibit pathological excitatory pathways while strengthening endogenous inhibitory mechanisms. Within this framework, modulation of K2P channels represents a powerful approach to restore excitability balance and an important component of future multimodal therapies for chronic pain disorders.

Beyond sensory neurons, several K2P channels are expressed in non-neuronal cells involved in pain processing, including microglia, macrophages, Schwann cells, satellite glial cells, and keratinocytes. Among them, **THIK1** is by far the best-characterized, with compelling evidence demonstrating its essential role in regulating microglial membrane potential, process surveillance, inflammasome activation, and IL-1β release (Kim et al., 2026). Given the well-established contribution of activated microglia and pro-inflammatory cytokines to chronic pain, these findings strongly suggest that **THIK1** could indirectly influence pain signaling. However, despite this strong mechanistic rationale, no study has yet directly demonstrated that THIK1-dependent microglial functions translate into altered pain behaviors *in vivo*. Likewise, **TASK1**, **TASK3**, and **TREK1** have all been implicated in pain regulation and are also expressed in several immune cell populations, including macrophages and lymphocytes, where they regulate membrane potential, cell activation, and inflammatory responses (Immanuel et al., 2024; Bittner et al., 2009). While current evidence primarily supports a neuronal contribution to their effects on pain, their immunological functions suggest that part of their impact on nociception may arise indirectly through the modulation of inflammation. In addition, other K2P channels, such as **TWIK2**, have also been identified in immune cells, particularly macrophages, where they have been implicated in processes including potassium homeostasis, inflammasome activation, and host defense. However, despite these emerging immunological functions, no study has yet investigated whether **TWIK2** contributes to pain signaling or pain-related behaviors although its involvement in macrophage-mediated inflammation makes it an attractive candidate for indirectly modulating inflammatory pain (Huang et al., 2023; Huang et al., 2025). In addition, several K2P channels, including **TWIK1**, **TREK1** and **TRAAK**, have been identified in keratinocytes and Schwann cells, two non-neuronal cell types that are increasingly recognized as active participants in somatosensory processing through bidirectional communication with peripheral sensory nerve endings (Kang et al., 2007). Keratinocytes can detect noxious mechanical, thermal, and chemical stimuli and release mediators that modulate nociceptor excitability, while Schwann cells have recently emerged as specialized mechanosensory cells capable of directly transducing painful mechanical stimuli (Abdo et al., 2019; Ojeda-Alonso et al., 2024; Wei et al., 2019). Although these observations raise the possibility that K2P channels may regulate pain through these non-neuronal cell populations, direct functional evidence supporting such a role is still lacking. Overall, despite the widespread expression of K2P channels in non-neuronal cells, their contribution to pain regulation remains largely unexplored and warrants further investigation using cell type-specific genetic and pharmacological approaches.

## Glossary

ACC: Anterior cingulate cortex
AITC: Allyl isothiocyanate
ASIC: Acid-sensing ion channel
AT1/2R: Angiotensin II type 1 or 2receptor
ATP: Adenosine triphosphate
CaV: Voltage-gated calcium channel
CCI: Chronic constriction injury
CFA: Complete Freund’s adjuvant
CGN: Cerebellar granular neurons
CGRP: Calcitonin gene-related peptide
CIPN: Chemotherapy-induced peripheral neuropathy
DNMT3: DNA methyltransferase 3
DPNP: Diabetic peripheral neuropathic pain
DRG: Dorsal root ganglion
FHEIG: Facial dysmorphism, Hypertrichosis, Epilepsy, Intellectual disability, Gingival overgrowth
**GABA**_**B**_: Gamma-aminobutyric acid type B receptor
GDNF: Glial cell line-derived neurotrophic factor
GOF: Gain-of-function
GPCR: G protein-coupled receptor
GRP: Gastrin-releasing peptide
GTEx: Genotype-tissue expression
HEK: Human embryonic kidney
IBA1: Ionized calcium-binding adapter molecule 1
IB4: Isolectin B4
ISDN: Isosorbide dinitrate
K2P: Two-pore-domain potassium channel
KIR: Inwardly rectifying potassium channel
KD: Knockdown
KO: Knockout
KV: Voltage-gated potassium channel
LOF: Loss-of-function
LPS: Lipopolysaccharide
LTMR: Low-Threshold Mechanoreceptor
MAPK: Mitogen-activated protein kinase
MODY: Maturity-onset diabetes of the young
MrgprA3: Mas-related G protein-coupled receptor A3
NaV: Voltage-gated sodium channel
NF200: Neurofilament 200
NLRP3: NOD-(nucleotide-binding oligomerization domain), LRR-(leucine-rich repeat) and pyrin domain-containing protein 3
NMB: Neuromedin B
NO: Nitric oxide
OPC: Oligomeric proanthocyanidins (also called oligomeric procyanidins)
P2XR: Purinergic P2X receptor
PAG: Periaqueductal gray
PGE2: Prostaglandin E2
PKA: Protein kinase A
PKC: Protein kinase C
PLC: Phospholipase C
PSNL: Partial sciatic nerve ligation
qPCR: quantitative polymerase chain reaction
RCAN1: Regulator of calcineurin 1
RET: Rearranged during transfection receptor tyrosine kinase
RMP: Resting membrane potential
RVM: Rostral ventromedial medulla
shRNA: Short hairpin RNA
siRNA: Small interfering RNA
SNA: Spinal nerve axotomy
SNI: Spared nerve injury
SNL: Spinal nerve ligation
SMRT: Single molecule real-time
SNP: Single nucleotide polymorphism
STOML3: Stomatin-like protein 3
TALK: TWIK-related alkaline pH-activated K^+^ channel
TASK: TWIK-related acid-sensitive K^+^ channel
TNBS: 2,4,6-trinitrobenzene sulfonic acid
TG: Trigeminal ganglion
THIK: Tandem-pore domain halothane-inhibited K^+^ channel
TOK: Tandem-pore outward rectifying K^+^ channel
TRAAK: TWIK-related arachidonic acid-stimulated K^+^ channel
TREK: TWIK-related K^+^ channel
TRESK: TWIK-related spinal cord K^+^ channel
TRP: Transient receptor potential
TRPA1: Transient receptor potential ankyrin 1
TRPM8: Transient receptor potential melastatin 8
TRPV1: Transient receptor potential vanilloid 1
TrkA: Tropomyosin receptor kinase A
TWIK: Tandem of pore domains in a weak inwardly rectifying K^+^ channel
UV: Ultraviolet
VEGF: Vascular endothelial growth factor
VEGFR: Vascular endothelial growth factor receptor
WT: Wild type
